# Sociodemographic Determinants and Temporal Trends in Periodontal Conditions in Brazil: A Population‐Based Cross‐Sectional Study

**DOI:** 10.1155/ijod/9047176

**Published:** 2026-07-18

**Authors:** Phelipe Elias da Silva, Lucas Gonçalves de Sousa, Heitor Bernardes Pereira Delfino, Catarina Machado Azeredo, Álex Moreira Herval, Mauro Henrique Nogueira Guimarães de Abreu, Priscilla Barbosa Ferreira Soares

**Affiliations:** ^1^ Department of Periodontology and Implantology, Faculty of Dentistry, Federal University of Uberlandia, Uberlandia, Brazil, ufu.br; ^2^ Department of Public Health and Forensic Dentistry, Faculty of Dentistry, Federal University of Uberlandia, Uberlandia, Brazil, ufu.br; ^3^ Department of Public Health, Faculty of Medicine, Federal University of Uberlandia, Uberlandia, Brazil, ufu.br; ^4^ Department of Community and Preventive Dentistry, Faculty of Dentistry, Federal University of Minas Gerais, Belo Horizonte, Brazil, ufmg.br

**Keywords:** dental health surveys, health status disparities, oral health, periodontal diseases

## Abstract

**Objectives:**

This study examined associations between sociodemographic characteristics and periodontal conditions in the Brazilian population across adolescents, adults, and older adults, and assessed temporal changes over a 13‐year period.

**Methods:**

This descriptive cross‐sectional study used data from the SB Brasil 2010 and 2023 national oral health surveys. Adolescents (15–19 years), adults (35–44 years), and older adults (65–74 years) with ≥20 natural teeth were included. Gingival signs, periodontal involvement, and its severe form were defined using standardized epidemiological thresholds. Weighted regression models accounting for the complex multistage sampling design and survey weights of the SB Brasil surveys were used to estimate associations and temporal differences.

**Results:**

Higher levels of educational attainment were inversely associated with severe periodontal involvement among older adults (odds ratio [OR] = 0.14; 95% confidence interval [CI]: 0.05–0.39). Women showed 35% lower odds of periodontal involvement and 58% lower odds of its severe form. Adolescents had approximately threefold higher odds of gingival signs, but 88% lower odds of periodontal involvement and 95% lower odds of severe periodontal conditions compared with older adults. Among adolescents, Black individuals had more than fourfold higher odds of periodontal involvement than their White counterparts. Between 2010 and 2023, this condition decreased in the overall population, particularly among adults, women, mixed‐race individuals, and those with intermediate levels of education, whereas the prevalence of gingival signs increased among adults.

**Conclusions:**

Patterns of periodontal conditions varied according to age and disease severity. While the prevalence of more advanced periodontal conditions was lower in 2023 than in 2010, early inflammatory conditions remained common. These findings highlight the importance of preventive strategies that address behavioral factors and broader social determinants of oral health.

## 1. Introduction

Periodontal diseases are among the most prevalent chronic conditions worldwide and represent a major public health challenge [1]. Recent estimates indicate that more than 1 billion people are affected globally, with continuing increases in both incidence and prevalence highlighting the magnitude and persistence of this burden [2]. Advanced periodontitis remains a leading cause of tooth loss in adults and is associated with substantial functional and social consequences, including impaired masticatory function and reduced quality of life [3, 4].

The distribution of periodontal conditions is heterogeneous and results from the interaction of biological, socioeconomic, and environmental factors [5, 6]. Sociodemographic characteristics influence exposure to risk factors, oral hygiene practices, and access to preventive and therapeutic dental services [7]. Consequently, higher prevalence rates are often observed among individuals living in socially and economically disadvantaged circumstances [8–10]. However, the extent to which socioeconomic inequalities affect different stages of periodontal disease remains uncertain.

In Brazil, the incorporation of oral health as a structural component of the Unified Health System, particularly following the implementation of the Brasil [11] Sorridente program, expanded epidemiological surveillance and monitoring of oral health conditions. National SB Brasil surveys allow for the monitoring of trends in oral diseases and related inequalities over time. These data provide a basis for updating estimates of periodontal diseases and for evaluating the effects of public policies, as well as demographic and socioeconomic changes [5, 12].

Recent evidence indicates that the burden of periodontal diseases varies according to social determinants, reinforcing the need for analyses stratified by sociodemographic characteristics [13]. In the Brazilian context, gaps persist in tracking temporal changes in these conditions across different population strata. This limitation restricts the evaluation of the impact of oral health interventions [14]. Integrating analytical approaches that consider these dimensions is therefore essential for identifying vulnerable groups and guiding public policies in periodontal health [10].

This study examined the associations between sociodemographic characteristics and periodontal conditions in the Brazilian population and across specific age groups—adolescents, adults, and older adults—using data from the SB Brasil 2023 survey. Additionally, temporal changes in periodontal conditions were assessed using comparable prevalence estimates from the SB Brasil 2010 and 2023 surveys to explore whether patterns of disease distribution across population groups have shifted over time.

## 2. Methods

### 2.1. Study Design and Population

This analytical cross‐sectional study used data from the Brazilian National Oral Health Survey (SB Brasil 2023), a nationally representative survey based on a multistage probabilistic sampling design covering the five major geographic regions of Brazil. For temporal comparisons, data from the SB Brasil 2010 survey were also included. Both surveys adopted comparable sampling strategies and clinical examination protocols, enabling direct comparisons between survey waves.

The study followed the principles of the Declaration of Helsinki. The SB Brasil 2010 and SB Brasil 2023 surveys were approved by the Brazilian National Research Ethics Committee (CONEP) (protocol numbers 15,498 and 4,823,054, respectively). Written informed consent was obtained from all participants aged 18 years or older, while consent for participants under 18 years of age was provided by their parents or legal guardians. The present study was based exclusively on anonymized secondary data from these surveys and therefore did not require additional review by the CEP/CONEP system, in accordance with Resolution No. 510/2016 of the Brazilian National Health Council.

### 2.2. Sampling and Data Collection

Participants were selected through multistage probabilistic sampling procedures representative of individuals living in permanent private households in state capitals and noncapital municipalities [15, 16]. In each survey wave, census tracts, households, and individuals were randomly selected within predefined age groups.

Data collection included household interviews and clinical examinations conducted by trained and calibrated teams. Interexaminer agreement was assessed using the weighted Kappa coefficient (0.65 in 2010 and 0.61 in 2023), indicating acceptable but not optimal reliability. Interviews obtained information on sociodemographic characteristics, dental service utilization, and other oral health determinants. Clinical examinations were performed according to World Health Organization (WHO) guidelines.

No additional sample size calculation was performed because the complete survey datasets were analyzed. Sampling weights were incorporated in all analyses to account for the survey design and produce population‐level estimates.

### 2.3. Study Variables

Participants with complete information on the community periodontal index (CPI), periodontal attachment loss (PAL), and relevant sociodemographic variables were included. The study population comprised adolescents (15–19 years), adults (35– 44 years), and older adults (65–74 years), following the age groups defined in the survey. Records with missing, incomplete, or inconsistent periodontal data were excluded.

Analyses were restricted to individuals with 20 or more natural teeth to reduce potential bias related to edentulism and extensive tooth loss and to improve the stability of periodontal estimates in population‐based assessments [17–19]. This criterion ensured a sufficient number of teeth and periodontal sites for assessment, which is particularly relevant when using partial recording protocols, as their validity depends on site coverage [20].

This restriction may, however, lead to lower estimates of the periodontal disease burden, particularly among older adults and socially disadvantaged groups with higher levels of tooth loss. To assess the impact of this decision, supplementary analyses including all participants, regardless of the number of teeth, were conducted, allowing for comparison of estimates and evaluation of the consistency of the findings.

Probing depth and clinical attachment loss were measured using standardized periodontal probes under natural light conditions. Sociodemographic characteristics and dental service utilization were obtained through structured interviews. Measurement procedures were considered methodologically equivalent between the 2010 and 2023 surveys.

Periodontal outcomes were derived from variables recorded in the survey database, specifically the CPI and PIP. The CPI variable included categories corresponding to gingival bleeding (code 1), dental calculus (code 2), shallow periodontal pockets of 4–5 mm (code 3), and deep periodontal pockets ≥6 mm (code 4). The PIP variable classified attachment loss as follows: 4–5 mm (code 1), 6–8 mm (code 2), 9–11 mm (code 3), and ≥12 mm (code 4).

Based on these measures, three periodontal conditions were defined. Gingival signs were identified by the presence of gingival bleeding and/or dental calculus. Periodontal involvement was defined as the presence of shallow periodontal pockets (≥4 mm) and/or attachment loss ≥4 mm. Severe periodontal involvement was defined as the presence of deep periodontal pockets (≥6 mm) and/or attachment loss ≥6 mm.

Periodontal thresholds (≥4 and ≥6 mm) were defined based on their consistent use in epidemiological studies and population‐based surveys, where probing depths ≥4 mm are generally interpreted as indicators of periodontal involvement and ≥6 mm as reflecting more advanced disease severity [13, 21]. These cut‐offs align with international consensus reports and epidemiological classifications that combine CPI scores with periodontal measurements [13] and were adopted to ensure comparability across survey waves.

The CPI, recommended by the WHO, offers a standardized and feasible approach for large‐scale data collection, despite inherent limitations related to partial recording protocols. Although these operational definitions differ from the case definitions proposed by the 2017 World Workshop classification [22], they remain widely used in population‐based research. For this reason, the periodontal outcomes assessed in this study should be interpreted as epidemiological indicators rather than clinical diagnoses of periodontitis.

The independent variables were educational level, age group, sex, race/skin color, and household crowding. The educational level, derived from years of schooling, was categorized as 0–4, 5–8, 9–11, and ≥12 years and was defined as the primary exposure. Age group, sex, and race/skin color were treated as structural confounders. Household crowding (≤3, 4–6, ≥7 residents) was considered a potential mediator based on a previously developed causal diagram [23].

**Figure 1:**
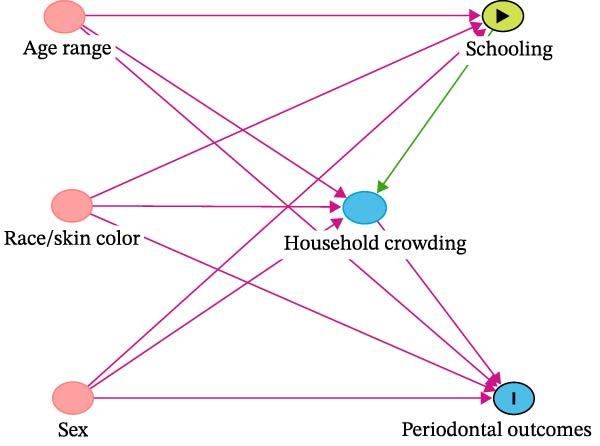
Directed acyclic graph (DAG) illustrating the relationships among sociodemographic characteristics, educational level, and periodontal outcomes. Age group, sex, and race/skin color act as structural determinants and confounders of the association between educational level (primary exposure) and periodontal outcomes. Household density was considered a potential mediator. The DAG guided the definition of the minimum adjustment set in the regression models.

To improve the representation of causal relationships, additional arrows were added between structural determinants to reflect plausible dependencies in the Brazilian social context. Evidence indicates that race/skin color, sex, and age influence educational opportunities and housing conditions, including household density patterns [24, 25]. These relationships were incorporated into the directed acyclic graph (DAG) (Figure 1) to more accurately represent the structural mechanisms preceding both the main exposure and the outcomes, and to inform the selection of covariates in the analytical models while minimizing inappropriate adjustment, particularly overadjustment and collider bias.

### 2.4. Statistical Analysis

Analyses of the SB Brasil 2023 dataset were conducted using IBM SPSS Statistics, version 31 (IBM Corp., Armonk, NY, USA), applying the complex samples module to account for the sampling design, including stratification, primary sampling units, and sampling weights. Sample characteristics were described using weighted proportions and corresponding 95% confidence intervals (95% CIs).

Associations between sociodemographic variables and periodontal outcomes were initially explored using weighted bivariate logistic regression models. Variables with *p*‐values < 0.25 were included in multivariable models. Adjusted odds ratios (ORs) and their respective 95% CIs were estimated using weighted logistic regression, with statistical significance assessed by the Wald test (*p* < 0.05).

These logistic regression models were applied to periodontal outcomes both restricted to individuals with ≥20 natural teeth and in the full sample, as well as to the outcome “fewer than 20 teeth” (supplementary analyses).

In addition to analyses of the overall population, models stratified by age groups were performed. The educational level was not included in adolescent models because schooling at this stage is strongly age‐dependent and may primarily reflect age progression rather than socioeconomic position [26]. Household crowding was included in supplementary models to explore potential living conditions‐related pathways between sociodemographic characteristics and periodontal outcomes.

Collinearity among independent variables was assessed using tolerance values and variance inflation factors (VIF). No relevant collinearity was identified (tolerance > 0.8 and VIF <2). Influential observations were examined using the Cook’s distance, with no values indicating excessive influence.

Logistic regression was used as the primary analytical approach due to its suitability for modeling binary outcomes and its ability to adjust for multiple covariates while accounting for the complex sampling design. ORs provide a valid measure of association in this context and do not rely on the rare disease assumption, although their interpretation differs from prevalence ratios (PRs) when outcomes are common [27].

Given previous evidence suggesting potential overestimation of associations when outcomes are frequent [28], additional models using Poisson regression with robust variance were fitted in Stata version 17.0 (StataCorp LLC, College Station, TX, USA) to estimate PRs, also accounting for the complex sampling design. These analyses were restricted to individuals with ≥20 natural teeth and included the same set of covariates as those in the main models. Results were compared to assess the consistency of the estimates.

Comparisons of periodontal outcome prevalence between the 2010 and 2023 surveys were performed using Stata version 17.0, accounting for the complex sampling design of both surveys through the svy commands. Weighted prevalences and corresponding 95% CIs were estimated for each survey year and stratified by sociodemographic variables. Absolute differences in prevalence between survey waves were estimated using Poisson regression models with an identity link function, including interaction terms between survey year and sociodemographic variables. Marginal prevalence differences and their respective 95% CIs were obtained from post‐estimation marginal analyses (margins).

## 3. Results

### 3.1. Participants

**Figure 2:**
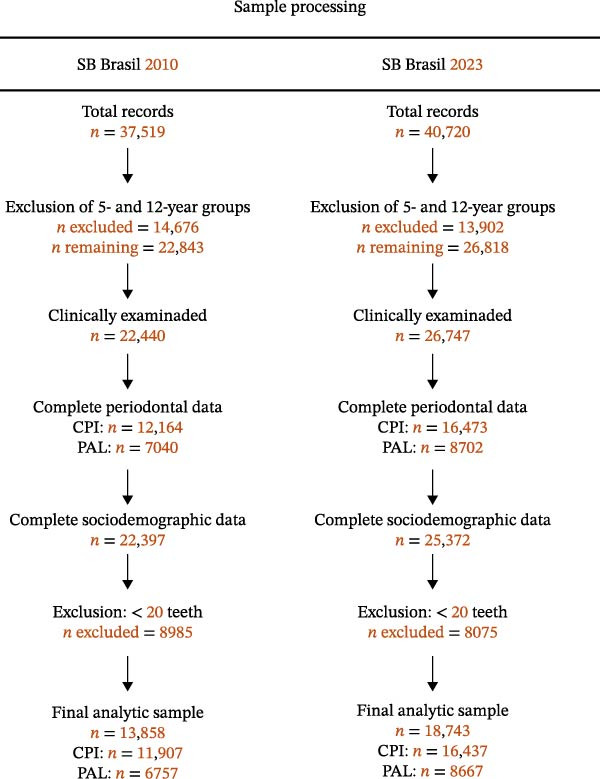
Flowchart of sample processing and selection (SB Brasil 2010 and 2023).

The SB Brasil surveys conducted in 2010 and 2023 included 37,519 and 40,720 examined participants, respectively. After applying the eligibility criteria—complete sociodemographic information, predefined age groups, complete periodontal examinations, and ≥20 natural teeth—the final analytical samples comprised 13,858 participants in 2010 and 18,743 in 2023. Figure 2 presents the flowchart of sample selection and exclusions, including participants with missing periodontal or sociodemographic information. Differences in the sample size across outcomes reflect the availability of valid periodontal assessments.

In 2010, the analytical sample was predominantly composed of adults and adolescents. In 2023, adults predominated, with a proportional reduction in adolescents and a slight increase in the participation of older adults compared with 2010. In both surveys, women and individuals self‐identified as White or mixed race were more frequently represented. Educational attainment differed between survey years, with a concentration in the 5–8‐ and 9–11‐years categories in 2010 and a predominance of 9–11 and ≥12 years in 2023. Household crowding showed a similar distribution across surveys, with most participants living in households with four to six residents. The prevalence of gingival signs was 33.3% in 2010 and 36.8% in 2023. Periodontal involvement affected 22.1% and 15.4% of participants, respectively. Disease severity remained infrequent in both surveys (5.3% and 4.0%). The overall sample characteristics are presented in Table 1.

**Table 1:** Characteristics of participants included in the analytical samples of the surveys.

Variables	2010 (*n* = 13,858)			2023 (*n* = 18,743)
	Frequency (unweighted)	Weighted (%)	Frequency (unweighted)	Weighted (%)
Demographic characteristics				
Age range				
Adolescents	5363	47.2	4930	27.3
Adults	7364	48.6	6149	64.3
Older adults	1131	4.1	1895	8.4
Sex				
Men	5630	43.5	5150	38.8
Women	8228	56.5	7824	61.2
Race/skin color				
White	6053	48.8	4371	43.9
Black	1507	10.7	1596	12.6
Asian	247	1.5	143	1.1
Brown/mixed race	5938	38.3	6687	42.2
Indigenous	113	0.7	47	0.3
Social characteristics				
Schooling				
0–4 years	1597	12.3	733	5.6
5–8 years	3927	28.1	1747	12.2
9–11 years	5221	40.0	6156	47.4
12 or more years	3039	19.6	4254	34.7
Household crowding				
Upto 3 people	4775	34.0	5675	46.7
4–6 people	7783	57.3	6603	50.1
7 or more people	1299	8.7	696	3.3
Periodontal outcomes				
Gingival signs				
Absent	9196	66.6	8148	63.2
Present	4662	33.4	4826	36.8
Periodontal involvement				
Absent	10,625	77.9	11249	84.6
Present	3233	22.1	1725	15.4
Severe periodontal involvement				
Absent	13,121	94.7	12606	96
Present	737	5.3	368	4

### 3.2. Associations Between Sociodemographic Characteristics and Periodontal Outcomes (SB Brasil 2023)

The findings for gingival signs are presented in Table 2. Adolescents and adults showed higher odds of gingival signs than older adults (OR: 3.53; 95% CI: 2.06–6.06 and OR: 2.96; 95% CI: 1.88–4.66, respectively). In age‐stratified analyses, Asian older adults showed lower odds than those of White individuals.

**Table 2:** Sociodemographic factors associated with gingival signs, SB Brasil 2023.

Categories (*n*)	Weighted (%)	Crude OR (95% CI)	*p*‐Value	Adjusted OR (95% CI)	Adjusted *p*‐value
All ages					
Schooling					
0–4 years (*n* = 170)	3.5	1	0.013	1	0.292
5–8 years (*n* = 593)	10	1.46 (0.86–2.49)		1.11 (0.62–1.97)	
9–11 years (*n* = 2446)	51	2.22 (1.30–3.80)		1.48 (0.81–2.71)	
12 or more years (*n* = 1599)	35.6	2.07 (1.20–3.54)		1.49 (0.81–2.73)	
Age range					
Older adults (*n* = 319)	3.5	1	<0.001	1	**<0.001**
Adults (*n* = 2388)	65.4	3.32 (2.14–5.15)	—	2.96 (1.88–4.66)	—
Adolescents (*n* = 2119)	31.2	4.01 (2.41–6.15)	3.53 (2.06–6.06)	—	—
Sex					
Men (*n* = 2010)	38.8	1	0.970	—	—
Women (*n* = 2816)	61.2	1.00 (0.83–1.20)		—	—
Race/skin color					
White (*n* = 1586)	42.2	1	0.600	—	—
Black (*n* = 610)	14.1	1.29 (0.79–2.09)		—	—
Asian (*n* = 49)	1.1	1.05 (0.48–2.31)		—	—
Brown/mixed race (*n* = 2524)	42.4	1.07 (0.86–1.34)		—	—
Indigenous (*n* = 14)	0.2	0.60 (0.26–1.39)		—	—
Older adults					
Schooling					
0–4 years (*n* = 63)	28.5	1	0.089	1	0.114
5–8 years (*n* = 74)	14.9	0.71 (0.27–1.91)	—	0.71 (0.26–1.92)	—
9–11 years (*n* = 74)	14.8	0.69 (0.27–1.79)	—	0.71 (0.27–1.95)	—
12 or more years (*n* = 106)	41.8	1.44 (0.47–4.45)	—	1.43 (0.49–4.60)	—
Sex					
Men (*n* = 130)	42.2	1	0.319	—	—
Women (*n* = 189)	57.8	1.37 (0.74–2.53)	—	—	—
Race/skin color					
White (*n* = 146)	63.9	1	0.013	1	**0.012**
Black (*n* = 50)	12.3	1.02 (0.51–2.05)	—	1.00 (0.50–1.97)	—
Asian (*n* = 3)	0.1	0.06 (0.01–0.38)	—	0.07 (0.01–0.37)	—
Brown/mixed race (*n* = 115)	23.5	0.72 (0.29–1.82)	—	0.74 (0.31–1.80)	—
Indigenous (*n* = 1)	0.3	3.59 (0.32–40.15)	—	3.78 (0.32–44.99)	—

*Note:* Estimates were obtained from logistic regression models accounting for the complex sampling design. Separate models were fitted for the overall population and for each age group (adolescents, adults, and older adults). Bold values indicate statistically significant associations (*p* < 0.05).

Abbreviations: CI, confidence interval; OR, odds ratio.

Adolescents were substantially less likely to present periodontal involvement than older adults (OR: 0.12; 95% CI: 0.07–0.20). Women also showed lower odds in the overall population (OR: 0.65; 95% CI: 0.50–0.84). In age‐stratified analyses, black adolescents showed higher odds of this outcome than White adolescents. Estimates for this condition are presented in Table 3.

**Table 3:** Sociodemographic correlates of periodontal involvement, SB Brasil 2023.

Categories (*n*)	Weighted (%)	Crude OR (95% CI)	*p*‐Value	Adjusted OR (95% CI)	Adjusted *p*‐value
All ages					
Schooling					
0–4 years (*n* = 138)	8.2	1	<0.001	1	0.061
5–8 years (*n* = 299)	19.1	1.10 (0.52–2.30)	—	1.40 (0.65–3.01)	—
9–11 years (*n* = 651)	39.8	0.51 (0.28–0.94)	—	0.92 (0.44–1.94)	—
12 or more years (*n* = 626)	32.9	0.59 (0.25–1.39)	—	0.72 (0.26–1.95)	—
Age range					
Older adults (*n* = 463)	14.3	1	<0.001	1	**<0.001**
Adults (*n* = 1039)	79.2	0.66 (0.47–0.92)	—	0.82 (0.50–1.35)	—
Adolescents (*n* = 223)	6.5	0.11 (0.07–0.17)	—	0.12 (0.07–0.20)	—
Sex					
Men (*n* = 745)	44.8	1	0.015	1	**0.001**
Women (*n* = 980)	55.2	0.75 (0.59–0.95)	—	0.65 (0.50–0.84)	—
Race/skin color					
White (*n* = 605)	46.3	1	0.153	1	0.157
Black (*n* = 266)	13.8	1.05 (0.60–1.85)	—	1.03 (0.54–1.97)	—
Asian (*n* = 18)	0.6	0.52 (0.19–1.40)	—	0.55 (0.19–1.63)	—
Brown/mixed race (*n* = 816)	38.6	0.85 (0.62–1.16)	—	0.91 (0.66–1.26)	—
Indigenous (*n* = 9)	0.6	2.48 (0.87–7.05)	—	2.80 (1.06–7.43)	—
Adolescents					
Sex					
Men (*n* = 119)	61.1	1	0.108	1	0.071
Women (*n* = 104)	38.9	0.61 (0.34–1.11)	—	0.58 (0.32–1.05)	—
Race/skin color					
White (*n* = 59)	29	1	0.004	1	**0.003**
Black (*n* = 30)	29	3.96 (1.64–9.56)	—	4.11 (1.67–10.09)	—
Asian (*n* = 4)	2.6	3.34 (0.61–18.43)	—	3.95 (0.75–20.72)	—
Brown/mixed race (*n* = 127)	39.4	1.09 (0.56–2.13)	—	1.10 (0.56–2.18)	—
Indigenous (*n* = 1)	0	0.15 (0.02–1.50)	—	0.16 (0.02–1.52)	—
Adults					
Schooling					
0–4 years (*n* = 48)	6.1	1	0.123	1	0.141
5–8 years (*n* = 160)	18.8	1.14 (0.43–3.04)	—	1.09 (0.44–2.70)	—
9–11 years (*n* = 400)	40.9	0.71 (0.29–1.75)	—	0.70 (0.29–1.67)	—
12 or more years (*n* = 429)	34.2	0.53 (0.16–1.73)	—	0.51 (0.16–1.63)	—
Sex					
Men (*n* = 403)	42	1	0.004	1	**0.005**
Women (*n* = 636)	58	0.62 (0.45–0.85)	—	0.63 (0.45–0.87)	—
Race/skin color					
White (*n* = 335)	45.2	1	0.067	1	0.052
Black (*n* = 163)	13.4	0.95 (0.50–1.97)	—	0.89 (0.41–1.91)	—
Asian (*n* = 9)	0.4	0.31 (0.09–1.14)	—	0.30 (0.08–1.09)	—
Brown/mixed race (*n* = 520)	40.2	0.94 (0.65–1.36)	—	0.87 (0.60–1.26)	—
Indigenous (*n* = 7)	0.8	3.23 (1.09–9.54)	—	3.12 (1.07–9.15)	—

*Note:* Estimates were obtained from logistic regression models accounting for the complex sampling design. Separate models were fitted for the overall population and for each age group (adolescents, adults, and older adults). Bold values indicate statistically significant associations (*p* < 0.05).

Abbreviations: CI, confidence interval; OR, odds ratio.

Destructive periodontal conditions showed distinct sociodemographic patterns (Table 4). Adolescents were less likely to present this condition than older adults (OR: 0.05; 95% CI: 0.02–0.13). Women also showed lower odds in the overall population (OR: 0.42; 95% CI: 0.23–0.74) and among adults (OR: 0.37; 95% CI: 0.20–0.68). Age‐stratified analyses indicated an inverse association between educational attainment and this outcome among older adults, with lower odds among individuals with higher levels of schooling. The full regression models are presented in Table S1.

**Table 4:** Sociodemographic correlates of severe periodontal involvement, SB Brasil 2023.

Categories (*n*)	Weighted (%)	Crude OR (95% CI)	*p*‐Value	Adjusted OR (95% CI)	Adjusted *p*‐value
All ages					
Schooling					
0–4 years (*n* = 52)	21.6	1	<0.001	1	0.088
5–8 years (*n* = 70)	9.8	0.18 (0.05–0.72)	—	0.20 (0.05–0.92)	—
9–11 years (*n* = 142)	47.9	0.23 (0.13–0.41)	—	0.40 (0.18–0.88)	—
12 or more years (*n* = 102)	20.8	0.14 (0.04–0.51)	—	0.16 (0.04–0.68)	—
Age range					
Older adults (*n* = 136)	17.7	1	<0.001	1	**<0.001**
Adults (*n* = 215)	79.7	0.56 (0.35–0.91)	—	1.06 (0.45–2.51)	—
Adolescents (*n* = 17)	2.5	0.04 (0.02–0.10)	—	0.05 (0.02–0.13)	—
Sex					
Men (*n* = 178)	54.8	1	0.020	1	**0.003**
Women (*n* = 190)	45.2	0.51 (0.29–0.90)	—	0.42 (0.23–0.74)	—
Race/skin color					
White (*n* = 124)	41.7	1	0.222	1	0.201
Black (*n* = 65)	10.8	0.90 (0.42–1.94)	—	0.82 (0.37–1.80)	—
Asian (*n* = 4)	0.7	0.64 (0.14–2.90)	—	0.70 (0.14–3.46)	—
Brown/mixed race (*n* = 168)	45.7	1.15 (0.57–2.31)	—	1.27 (0.65–2.50)	—
Indigenous (*n* = 3)	1.1	4.49 (1.11–18.19)	—	6.27 (1.24–31.74)	—
Adults					
Schooling					
0–4 years (*n* = 18)	15.9	1	0.103	1	0.082
5–8 years (*n* = 30)	7.2	0.14 (0.02–0.83)	—	0.12 (0.02–0.70)	—
9–11 years (*n* = 97)	53.2	0.34 (0.14–0.81)	—	0.33 (0.13–0.80)	—
12 or more years (*n* = 69)	23.7	0.14 (0.03–0.72)	—	0.13 (0.03–0.65)	—
Sex					
Men (*n* = 94)	54.5	1	0.004	1	**0.001**
Women (*n* = 121)	45.5	0.39 (0.21–0.74)	—	0.37 (0.20–0.68)	—
Race/skin color					
White (*n* = 58)	39.6	1	0.102	1	0.175
Black (*n* = 38)	10.4	0.85 (0.32–2.27)	—	0.72 (0.27–1.96)	—
Asian (*n* = 2)	0.5	0.48 (0.06–3.90)	—	0.42 (0.05–3.77)	—
Brown/mixed race (*n* = 111)	48.2	1.32 (0.67–2.60)	—	1.27 (0.60–2.67)	—
Indigenous (*n* = 3)	1.4	5.72 (1.33–24.64)	—	7.56 (1.27–45.02)	—
Older adults					
Schooling					
0–4 years (*n* = 33)	53.2	1	0.001	1	**0.001**
5–8 years (*n* = 37)	20.5	0.51 (0.18–1.45)	—	0.51 (0.18–1.45)	—
9–11 years (*n* = 32)	17	0.41 (0.15–1.12)	—	0.41 (0.15–1.12)	—
12 or more years (*n* = 33)	9.2	0.14 (0.05–0.39)	—	0.14 (0.05–0.39)	—
Sex					
Men (*n* = 76)	58.2	1	0.390	—	—
Women (*n* = 60)	41.8	0.66 (0.26–1.70)	—	—	—
Race/skin color					
White (*n* = 60)	50.2	1	—	—	—
Black (*n* = 25)	13.1	1.42 (0.45–4.45)	—	—	—
Asian (*n* = 2	1.7	1.48 (0.11–20.36)	—	—	—
Brown/mixed race (*n* = 48)	35	1.49 (0.46–4.84)	—	—	—
Indigenous (*n* = 0)	—	—	—	—	—

*Note:* Estimates were obtained from logistic regression models accounting for the complex sampling design. Separate models were fitted for the overall population and for each age group (adolescents, adults, and older adults). Bold values indicate statistically significant associations (*p* < 0.05).

Abbreviations: CI, confidence interval; OR, odds ratio.

In supplementary analyses including household crowding as a proxy of living conditions (Table S2), higher odds of periodontal involvement were observed among adolescents living in households with four to six residents than among those with up to three residents.

Given the potential underrepresentation of individuals with advanced tooth loss due to the ≥20 teeth restriction, supplementary analyses were conducted to evaluate its impact. When “fewer than 20 teeth” was considered as the outcome (Table S3), older adults were more likely to present reduced dentition, with an inverse association with higher educational attainment observed in the overall population (OR: 0.13; 95% CI: 0.07–0.22), as well as among adults and older adults. Older women also showed higher odds (OR: 1.96; 95% CI: 1.47–2.60).

Repeating the periodontal analyses in the full sample, regardless of the number of teeth (Table S4), yielded largely consistent results, with some differences. These included a positive association between higher educational attainment and gingival signs (OR = 2.47; 95% CI: 1.42–4.30), as well as higher odds of periodontal outcomes among adults (OR = 2.61; 95% CI: 1.50–4.55). Among older adults, higher educational attainment was associated with periodontal involvement (OR = 4.10; 95% CI: 2.06–8.13) but not with its severe form; however, these estimates should be interpreted with caution given the width of the CIs. In addition, older women consistently showed lower odds across periodontal involvement (OR: 0.51; 95% CI: 0.34–0.76).

ORs should be interpreted with caution given the relatively high prevalence of some outcomes; therefore, additional analyses using Poisson regression restricted to individuals with 20 or more teeth were performed. These analyses showed similar results, supporting the robustness of the estimates (Table S5). Differences were limited to a lower prevalence of periodontal involvement among adults with higher educational attainment (PR: 0.16; 95% CI: 0.04–0.59).

### 3.3. Temporal Variation in Periodontal Conditions Between 2010 and 2023

**Figure 3:**
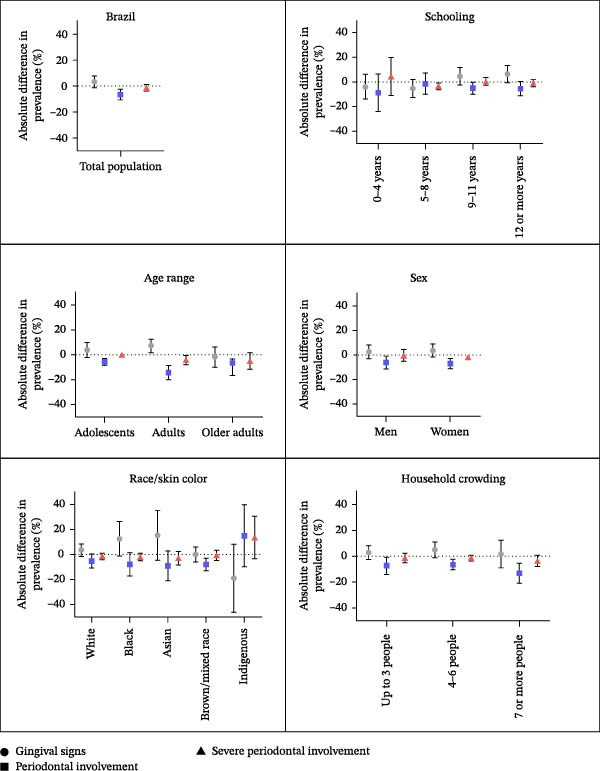
Differences in the prevalence of periodontal outcomes between 2010 and 2023 in the Brazilian population. Points represent prevalence differences (percentage points) and error bars indicate 95% confidence intervals. Estimates with confidence intervals crossing zero indicate no clear change between survey waves.

Temporal comparisons between the 2010 and 2023 surveys are presented in Figure 3 and Table S6. Over the study period, a significant reduction in periodontal involvement prevalence was observed in the overall population (−6.68 percentage points [p.p.]), whereas no statistically significant changes were detected for the other outcomes.

Temporal differences varied by age groups. Among adults, the prevalence of gingival signs increased (+7.51 p.p.), while periodontal involvement (−14.41 p.p.) and destructive periodontal conditions (−4.17 p.p.) declined. Among adolescents, a significant reduction was observed only for periodontal involvement (−5.83 p.p.).

According to sex, women showed reductions in periodontal involvement (−6.90 p.p.) and its severe form (−1.86 p.p.), whereas among men, the reduction was limited to periodontal status (−5.99 p.p.).

Regarding race/skin color, a significant reduction was observed only for periodontal involvement among individuals self‐identified as mixed‐race (−8.04 p.p.). By educational level, individuals with 9–11 years of schooling showed a reduction in periodontal condition (−4.99 p.p.), whereas those with 5–8 years showed a decrease in its severe form (−3.63 p.p.).

Finally, considering household crowding, periodontal involvement declined across all evaluated strata.

## 4. Discussion

This study examined the associations between sociodemographic characteristics and periodontal conditions in a nationally representative sample of the Brazilian population, as well as temporal changes observed between the SB Brasil 2010 and 2023 surveys. The findings indicate that the social determinants of health do not operate uniformly across periodontal outcomes. Instead, distinct patterns emerged according to the type of condition and age group. These results underscore the importance of analytical approaches that are sensitive to age‐related heterogeneity and to the specific nature of periodontal outcomes.

The educational attainment, the main exposure in this study, was not related to gingival signs or periodontal involvement in the overall population when analyses were restricted to individuals with ≥20 teeth. When this restriction was not applied, higher educational attainment was positively related to gingival signs, possibly reflecting greater tooth retention and a higher number of sites at risk for gingival inflammation, as well as increased detection of early inflammatory conditions [29]. This pattern supports the view that gingival inflammation reflects more immediate, behavior‐related exposures, being less dependent on accumulated educational capital [30, 31] and may therefore occur relatively uniformly across social strata.

In contrast, higher education was associated with a lower prevalence of reduced dentition (<20 teeth) among adults and older adults, as well as with reduced levels of severe periodontal involvement among older adults with ≥20 teeth. These findings are consistent with patterns in which educational attainment is associated with more severe and cumulative outcomes, consistent with evidence that destructive periodontal conditions reflect long‐term social exposures [32, 33], while greater access to resources and care among more educated individuals may limit disease progression over the life course [14, 34, 35].

Age‐specific associations further reinforce this interpretation. Adolescents showed a higher likelihood of gingival signs but lower odds of periodontal involvement and its severe form compared with older adults. This pattern is consistent with the natural history of periodontal disease, in which gingivitis represents an early and potentially reversible inflammatory process [36, 37]. Such conditions are common during life stages marked by irregular oral hygiene practices and lower risk perceptions [38, 39]. In contrast, the low frequency of severe periodontal conditions among adolescents likely reflects insufficient cumulative exposure to etiological factors required for advanced periodontal destruction [40, 41].

Sex‐related differences showed a consistent pattern of lower odds of periodontal involvement and destructive periodontal conditions among women, both in the overall population and across age strata. This finding is well documented in the literature and has been largely attributed to the greater use of preventive services and more regular self‐care practices among women [42, 43]. In contrast, men tend to use dental services less frequently, have lower oral health literacy, and show less consistent adherence to preventive behaviors [42, 44, 45].

However, older women showed a higher likelihood of reduced dentition (<20 teeth), which may reflect cumulative life‐course influences and cohort effects, including historical treatment patterns [46]. Hormonal changes associated with menopause may also have contributed [47, 48], although these mechanisms cannot be directly assessed.

Overall, these findings suggest that tooth loss and current periodontal conditions capture distinct processes, with older women showing greater tooth loss but lower odds of periodontal involvement than those of men.

This distinction is particularly relevant among older adults, in whom cumulative tooth loss may partly reflect previous periodontal destruction occurring earlier in life [46, 49]. As a result, individuals with the most severe historical disease burden may be underrepresented in analyses of current periodontal conditions [50, 51], even when supplementary analyses including participants with fewer than 20 teeth are considered. This highlights the importance of interpreting periodontal outcomes and tooth loss as complementary indicators of oral health across the life course [5].

Associations according to race/skin color were observed only in specific age groups. Higher odds of periodontal involvement were found among adolescents self‐identified as black compared with those of white individuals. These findings should be interpreted within a structural framework of health determinants. In the Brazilian context, race/skin color operates as a marker of historically constructed social inequalities [52], including disparities in access to education [53], income [54], housing conditions [55], and health services [56, 57].

The supplementary analysis incorporating household density as a proxy of living conditions identified an association only for more advanced periodontal conditions among adolescents living in households with four to six residents. Although exploratory, this finding suggests that material household conditions may play a role even at the early stages of life, possibly through mechanisms related to overcrowding, resource sharing, and greater exposure to environments unfavorable to health [58, 59]. The wide CI warrants cautious interpretation and highlights the need for future studies using more detailed measures of the housing context.

Regarding temporal trends, a significant decline in the prevalence of periodontal involvement was observed between 2010 and 2023, particularly among adults and women, whereas gingival signs remained stable or increased in specific population strata. This pattern may reflect improvements in the management and control of more advanced forms of periodontal disease, potentially associated with the expansion of primary oral healthcare coverage and the consolidation of public policies such as the Brasil Sorridente program [60].

Data from SB Brasil 2023 indicate that most adults reported using dental services in both the public and private sectors [61], which may have contributed to the earlier diagnosis and treatment of severe cases. Additionally, a reduction in DMFT was observed across the SB Brasil 2003, 2010, and 2023 surveys among adolescents, highlighting consistent advances in oral health conditions in this age group [62].

Part of the observed differences between survey waves may also reflect broader demographic and social changes that occurred in Brazil during the study period [63, 64]. Improvements in educational attainment, changes in population age structure, and expansions in access to health and social services may have influenced periodontal outcomes independently of changes in oral healthcare delivery [63–66]. Therefore, the temporal differences observed should be interpreted within the context of these wider societal transformations.

Despite these advances, structural and organizational barriers persist, including limitations in the performance of Dental Specialty Centers [67] and a recent decline in the provision of specialized dental procedures [68]. Moreover, in 2022, only 62.3% of individuals who sought dental care within the public system were actually treated, highlighting ongoing gaps in access [61]. In contrast, the stable or increasing prevalence of gingival signs points to weaknesses in current preventive strategies, particularly those aimed at promoting daily oral hygiene practices and primary prevention among younger populations [12, 69].

Longitudinal studies are needed to better elucidate the causal pathways linking educational attainment, living conditions, and the progression of periodontal disease. Future research incorporating more detailed measures of the housing context and access to dental care may provide a more comprehensive understanding of the mechanisms underlying social inequalities in periodontal health.

Some limitations should be considered. Given the cross‐sectional design, temporal ordering between exposure and outcomes cannot be established, and the observed associations should not be interpreted as causal relationships. In addition, although the CPI recommended by the WHO enables standardized data collection, its limitations are well documented [70].

The use of partial‐mouth recording protocols based on index teeth may reduce sensitivity and introduce measurement bias [71, 72]. In addition, CPI has limited ability to discriminate disease severity [73], and variability in examination protocols may affect comparability across studies [74].

Temporal comparisons were descriptive and did not account for potential shifts in population composition between the survey waves. Broader demographic, educational, and social changes that occurred in Brazil between 2010 and 2023 may also have influenced periodontal outcomes independently of changes in oral healthcare delivery.

Because periodontal analyses were restricted to individuals with at least 20 teeth, some degree of dental survival bias cannot be excluded, particularly among older adults, as individuals with a history of more severe periodontal destruction may already have lost the affected teeth. Although supplementary analyses were performed to assess the impact of this restriction, the possibility of residual bias remains.

Finally, some stratified analyses involved a limited number of participants in specific race/skin color categories, particularly indigenous and Asian individuals, resulting in wide CIs and lower statistical precision. These estimates should therefore be interpreted with caution.

Despite these limitations, the study benefits from its national representativeness, methodological comparability between survey waves, appropriate handling of the complex sampling design, and stratified analyses by age groups.

## 5. Conclusions

These findings suggest a reduction in some periodontal outcomes between 2010 and 2023, particularly periodontal involvement, although changes were not observed uniformly across all population groups and conditions evaluated. Early inflammatory periodontal conditions remained highly prevalent, highlighting the persistence of factors associated with periodontal inflammation. Strengthening preventive strategies within primary oral healthcare, particularly those focused on younger individuals and socially vulnerable populations, may help address behavioral risk factors and reduce inequalities in periodontal health. Integrating preventive oral health actions with broader policies targeting social determinants may further contribute to improving periodontal health throughout the life course.

## Author Contributions

Lucas Gonçalves de Sousa, Heitor Bernardes Pereira Delfino, Catarina Machado Azeredo, Álex Moreira Herval, Mauro Henrique Nogueira Guimarães de Abreu, and Priscilla Barbosa Ferreira Soares conceptualized the study. Phelipe Elias da Silva performed the statistical analyses and drafted the manuscript. All authors have contributed to the interpretation of the findings and critically revised the manuscript.

## Funding

This work was supported by the Coordenação de Aperfeiçoamento de Pessoal de Nível Superior ( CAPES ) (Finance Code 001), the Conselho Nacional de Desenvolvimento Científico e Tecnológico (CNPq) (Grants 407933/2021‐2 and 313491/2021‐6), the INCT–Saúde Oral e Odontologia (Grant 406840/2022‐9), and the Fundação de Amparo à Pesquisa do Estado de Minas Gerais ( FAPEMIG ) (Grants APQ‐03238‐24 and RED‐00204‐23).

## Disclosure

All authors have approved the final version of the manuscript.

## Conflicts of Interest

The authors declare no conflicts of interest.

## Supporting Information

Additional supporting information can be found online in the Supporting Information section.

## Supporting information


**Supporting Information** Supporting Information are provided in a separate file and include three tables presenting the full regression models, additional association analyses, and weighted prevalences and temporal changes in periodontal outcomes. Table S1. Fully adjusted weighted logistic regression models for gingival signs, periodontal involvement, and severe periodontal involvement among individuals with ≥20 natural teeth (SB Brasil 2023). Table S2. Associations between sociodemographic characteristics, including household crowding, and periodontal outcomes in the overall population and by age group (SB Brasil 2023). Table S3. Weighted logistic regression models for fewer than 20 teeth (SB Brasil 2023). Table S4. Weighted logistic regression analyses of periodontal outcomes in the full sample, regardless of the number of teeth (SB Brasil 2023). Table S5. Fully adjusted weighted Poisson regression models for gingival signs, periodontal involvement, and severe periodontal involvement among individuals with ≥20 natural teeth (SB Brasil 2023). Table S6. Weighted prevalences and temporal changes in periodontal outcomes between 2010 and 2023 according to sociodemographic characteristics (2023–2010).

## Data Availability

The data that support the findings of this study are available from the corresponding author upon reasonable request.
