# Supplementary material for: Sociodemographic Determinants and Temporal Trends in Periodontal Conditions in Brazil: A Population‐Based Cross‐Sectional Study
Source: Int J Dent. 2026 Jul 18;2026:9047176. doi: 10.1155/ijod/9047176 (PMC13379951; doi:10.1155/ijod/9047176)
Supplement: Supplementary file 1 — Supporting Information Supporting Information are provided in a separate file and include three tables presenting the full regression models, additional association analyses, and weighted prevalences and temporal changes in periodontal outcomes. Table S1. Fully adjusted weighted logistic regression models for gingival signs, periodontal involvement, and severe periodontal involvement among individuals with ≥20 natural teeth (SB Brasil 2023). Table S2. Associations between sociodemographic characteristics, including household crowding, and periodontal outcomes in the overall population and by age group (SB Brasil 2023). Table S3. Weighted logistic regression models for fewer than 20 teeth (SB Brasil 2023). Table S4. Weighted logistic regression analyses of periodontal outcomes in the full sample, regardless of the number of teeth (SB Brasil 2023). Table S5. Fully adjusted weighted Poisson regression models for gingival signs, periodontal involvement, and severe periodontal involvement among individuals with ≥20 natural teeth (SB Brasil 2023). Table S6. Weighted prevalences and temporal changes in periodontal outcomes between 2010 and 2023 according to sociodemographic characteristics (2023–2010). [file IJOD-2026-9047176-s001.docx]

**SUPPLEMENTARY MATERIAL**

**Supplementary Table 1.** Fully adjusted weighted logistic regression models for gingival signs, periodontal involvement, and severe periodontal involvement among individuals with ≥20 natural teeth (SB Brasil 2023).

| **Outcome** | **Sociodemographic variables** | **Categories**  **(n)** | **Weighted**  **(%)** | **Crude OR^a^**  **(95% CI^b^)** | **p-value** | **Adjusted OR**  **(95% CI)** | **Adjusted p-value** |
| --- | --- | --- | --- | --- | --- | --- | --- |
| Gingival signs | ***All ages*** | | | | | | |
|  | Schooling | 0-4 years (n=170) | 3.5% | 1 | 0.013 | 1 | 0.292 |
|  |  | 5-8 years (n=593) | 10% | 1.46 (0.86-2.49) |  | 1.11 (0.62-1.97) |  |
|  |  | 9-11 years (n=2446) | 51% | 2.22 (1.30-3.80) |  | 1.48 (0.81-2.71) |  |
|  |  | 12 or more years (n=1599) | 35.6% | 2.07 (1.20-3.54) |  | 1.49 (0.81-2.73) |  |
|  | Age range | Older adults (n=319) | 3.5% | 1 | <0.001 | 1 | **<0.001** |
|  |  | Adults (n=2388) | 65.4% | 3.32 (2.14-5.15) |  | 2.96 (1.88-4.66) |  |
|  |  | Adolescents (n=2119) | 31.2% | 4.01 (2.41-6.15) |  | 3.53 (2.06-6.06) |  |
|  | Sex | Men (n=2010) | 38.8% | 1 | 0.970 | -- | -- |
|  |  | Women (n=2816) | 61.2% | 1.00 (0.83-1.20) |  |  |  |
|  | Race/Skin color | White (n=1586) | 42.2% | 1 | 0.600 | -- | -- |
|  |  | Black (n=610) | 14.1% | 1.29 (0.79-2.09) |  |  |  |
|  |  | Asian (n=49) | 1.1% | 1.05 (0.48-2.31) |  |  |  |
|  |  | Brown/Mixed race (n=2524) | 42.4% | 1.07 (0.86-1.34) |  |  |  |
|  |  | Indigenous (n=14) | 0.2% | 0.60 (0.26-1.39) |  |  |  |
|  | ***Adolescents*** | | | | | | |
|  | Sex | Men (n=1098) | 51.9% | 1 | 0.342 | -- | -- |
|  |  | Women (n=1021) | 48.1% | 0.85 (0.60-1.19) |  |  |  |
|  | Race/Skin color | White (n=649) | 42.2% | 1 | 0.866 | -- | -- |
|  |  | Black (n=242) | 14.1% | 1.16 (0.68-2.00) |  |  |  |
|  |  | Asian (n=21) | 1.1% | 0.75 (0.24-2.33) |  |  |  |
|  |  | Brown/Mixed race (n=1180) | 42.4% | 0.95 (0.66-1.37) |  |  |  |
|  |  | Indigenous (n=8) | 0.2% | 1.59 (0.17-14.96) |  |  |  |
|  | ***Adults*** | | | | | | |
|  | Schooling | 0-4 years (n=84) | 3.3% | 1 | 0.325 | -- | -- |
|  |  | 5-8 years (n=266) | 10.8% | 1.24 (0.56-2.75) |  |  |  |
|  |  | 9-11 years (n=860) | 42.8% | 1.80 (0.79-4.10) |  |  |  |
|  |  | 12 or more years (n=1168) | 43.2% | 1.66 (0.77-3.57) |  |  |  |
|  | Sex | Men (n=782) | 32.3% | 1 | 0.699 | -- | -- |
|  |  | Women (n=1606) | 67.4% | 1.05 (0.82-1.33) |  |  |  |
|  | Race/Skin color | White (n=791) | 42.4% | 1 | 0.374 | -- | -- |
|  |  | Black (n=318) | 15.4% | 1.31 (0.68-2.50) |  |  |  |
|  |  | Asian (n=25) | 1.2% | 1.35 (0.49-3.72) |  |  |  |
|  |  | Brown/Mixed race (n=1229) | 41% | 1.05 (0.75-1.48) |  |  |  |
|  |  | Indigenous (n=5) | 0.1% | 0.28 (0.07-1.16) |  |  |  |
|  | ***Older adults*** | | | | | | |
|  | Schooling | 0-4 years (n=63) | 28.5% | 1 | 0.089 | 1 | 0.114 |
|  |  | 5-8 years (n=74) | 14.9% | 0.71 (0.27-1.91) |  | 0.71 (0.26-1.92) |  |
|  |  | 9-11 years (n=74) | 14.8% | 0.69 (0.27-1.79) |  | 0.71 (0.27-1.95) |  |
|  |  | 12 or more years (n=106) | 41.8% | 1.44 (0.47-4.45) |  | 1.43 (0.49-4.60) |  |
|  | Sex | Men (n=130) | 42.2% | 1 | 0.319 | -- | -- |
|  |  | Women (n=189) | 57.8% | 1.37 (0.74-2.53) |  |  |  |
|  | Race/Skin color | White (n=146) | 63.9% | 1 | 0.013 | 1 | **0.012** |
|  |  | Black (n=50) | 12.3% | 1.02 (0.51-2.05) |  | 1.00 (0.50-1.97) |  |
|  |  | Asian (n=3) | 0.1% | 0.06 (0.01-0.38) |  | 0.07 (0.01-0.37) |  |
|  |  | Brown/Mixed race (n=115) | 23.5% | 0.72 (0.29-1.82) |  | 0.74 (0.31-1.80) |  |
|  |  | Indigenous (n=1) | 0.3% | 3.59 (0.32-40.15) |  | 3.78 (0.32-44.99) |  |
| Periodontal involvement | ***All ages*** | | | | | | |
|  | Schooling | 0-4 years (n=138) | 8.2% | 1 | <0.001 | 1 | 0.061 |
|  |  | 5-8 years (n=299) | 19.1% | 1.10 (0.52-2.30) |  | 1.40 (0.65-3.01) |  |
|  |  | 9-11 years (n=651) | 39.8% | 0.51 (0.28-0.94) |  | 0.92 (0.44-1.94) |  |
|  |  | 12 or more years (n=626) | 32.9% | 0.59 (0.25-1.39) |  | 0.72 (0.26-1.95) |  |
|  | Age range | Older adults (n=463) | 14.3% | 1 | <0.001 | 1 | **<0.001** |
|  |  | Adults (n=1039) | 79.2% | 0.66 (0.47-0.92) |  | 0.82 (0.50-1.35) |  |
|  |  | Adolescents (n=223) | 6.5% | 0.11 (0.07-0.17) |  | 0.12 (0.07-0.20) |  |
|  | Sex | Men (n=745) | 44.8% | 1 | 0.015 | 1 | **0.001** |
|  |  | Women (n=980) | 55.2% | 0.75 (0.59-0.95) |  | 0.65 (0.50-0.84) |  |
|  | Race/Skin color | White (n=605) | 46.3% | 1 | 0.153 | 1 | 0.157 |
|  |  | Black (n=266) | 13.8% | 1.05 (0.60-1.85) |  | 1.03 (0.54-1.97) |  |
|  |  | Asian (n=18) | 0.6% | 0.52 (0.19-1.40) |  | 0.55 (0.19-1.63) |  |
|  |  | Brown/Mixed race (n=816) | 38.6% | 0.85 (0.62-1.16) |  | 0.91 (0.66-1.26) |  |
|  |  | Indigenous (n=9) | 0.6% | 2.48 (0.87-7.05) |  | 2.80 (1.06-7.43) |  |
|  | ***Adolescents*** | | | | | | |
|  | Sex | Men (n=119) | 61.1% | 1 | 0.108 | 1 | 0.071 |
|  |  | Women (n=104) | 38.9% | 0.61 (0.34-1.11) |  | 0.58 (0.32-1.05) |  |
|  | Race/Skin color | White (n=59) | 29% | 1 | 0.004 | 1 | **0.003** |
|  |  | Black (n=30) | 29% | 3.96 (1.64-9.56) |  | 4.11 (1.67-10.09) |  |
|  |  | Asian (n=4) | 2.6% | 3.34 (0.61-18.43) |  | 3.95 (0.75-20.72) |  |
|  |  | Brown/Mixed race (n=127) | 39.4% | 1.09 (0.56-2.13) |  | 1.10 (0.56-2.18) |  |
|  |  | Indigenous (n=1) | 0% | 0.15 (0.02-1.50) |  | 0.16 (0.02-1.52) |  |
|  | ***Adults*** | | | | | | |
|  | Schooling | 0-4 years (n=48) | 6.1% | 1 | 0.123 | 1 | 0.141 |
|  |  | 5-8 years (n=160) | 18.8% | 1.14 (0.43-3.04) |  | 1.09 (0.44-2.70) |  |
|  |  | 9-11 years (n=400) | 40.9% | 0.71 (0.29-1.75) |  | 0.70 (0.29-1.67) |  |
|  |  | 12 or more years (n=429) | 34.2% | 0.53 (0.16-1.73) |  | 0.51 (0.16-1.63) |  |
|  | Sex | Men (n=403) | 42% | 1 | 0.004 | 1 | **0.005** |
|  |  | Women (n=636) | 58% | 0.62 (0.45-0.85) |  | 0.63 (0.45-0.87) |  |
|  | Race/Skin color | White (n=335) | 45.2% | 1 | 0.067 | 1 | 0.052 |
|  |  | Black (n=163) | 13.4% | 0.95 (0.50-1.97) |  | 0.89 (0.41-1.91) |  |
|  |  | Asian (n=9) | 0.4% | 0.31 (0.09-1.14) |  | 0.30 (0.08-1.09) |  |
|  |  | Brown/Mixed race (n=520) | 40.2% | 0.94 (0.65-1.36) |  | 0.87 (0.60-1.26) |  |
|  |  | Indigenous (n=7) | 0.8% | 3.23 (1.09-9.54) |  | 3.12 (1.07-9.15) |  |
|  | ***Older adults*** | | | | | | |
|  | Schooling | 0-4 years (n=83) | 24.4% | 1 | 0.816 | -- | -- |
|  |  | 5-8 years (n=101) | 19.7% | 1.21 (0.53-2.77) |  |  |  |
|  |  | 9-11 years (n=111) | 22.7% | 1.43 (0.65-3.15) |  |  |  |
|  |  | 12 or more years (n=160) | 33.1% | 1.34 (0.63-2.85) |  |  |  |
|  | Sex | Men (n=223) | 52.8% | 1 | 0.353 | -- | -- |
|  |  | Women (n=240) | 47.2% | 0.81 (0.51-1.27) |  |  |  |
|  | Race/Skin color | White (n=211) | 60.1% | 1 | 0.933 | -- | -- |
|  |  | Black (n=73) | 9.3% | 0.76 (0.35-1.65) |  |  |  |
|  |  | Asian (n=5) | 1.2% | 0.81 (0.09-7.43) |  |  |  |
|  |  | Brown/Mixed race (n=169) | 29.3% | 1.01 (0.54-1.89) |  |  |  |
|  |  | Indigenous (n=1) | 0.1% | 1.50 (0.13-16.77) |  |  |  |
| Severe periodontal involvement | ***All ages*** | | | | | | |
|  | Schooling | 0-4 years (n=52) | 21.6% | 1 | <0.001 | 1 | 0.088 |
|  |  | 5-8 years (n=70) | 9.8% | 0.18 (0.05-0.72) |  | 0.20 (0.05-0.92) |  |
|  |  | 9-11 years (n=142) | 47.9% | 0.23 (0.13-0.41) |  | 0.40 (0.18-0.88) |  |
|  |  | 12 or more years (n=102) | 20.8% | 0.14 (0.04-0.51) |  | 0.16 (0.04-0.68) |  |
|  | Age range | Older adults (n=136) | 17.7% | 1 | <0.001 | 1 | **<0.001** |
|  |  | Adults (n=215) | 79.7% | 0.56 (0.35-0.91) |  | 1.06 (0.45-2.51) |  |
|  |  | Adolescents (n=17) | 2.5% | 0.04 (0.02-0.10) |  | 0.05 (0.02-0.13) |  |
|  | Sex | Men (n=178) | 54.8% | 1 | 0.020 | 1 | **0.003** |
|  |  | Women (n=190) | 45.2% | 0.51 (0.29-0.90) |  | 0.42 (0.23-0.74) |  |
|  | Race/Skin color | White (n=124) | 41.7% | 1 | 0.222 | 1 | 0.201 |
|  |  | Black (n=65) | 10.8% | 0.90 (0.42-1.94) |  | 0.82 (0.37-1.80) |  |
|  |  | Asian (n=4) | 0.7% | 0.64 (0.14-2.90) |  | 0.70 (0.14-3.46) |  |
|  |  | Brown/Mixed race (n=168) | 45.7% | 1.15 (0.57-2.31) |  | 1.27 (0.65-2.50) |  |
|  |  | Indigenous (n=3) | 1.1% | 4.49 (1.11-18.19) |  | 6.27 (1.24-31.74) |  |
|  | ***Adolescents*** | | | | | | |
|  | Sex | Men (n=8) | 41.6% | 1 | 0.684 | -- | -- |
|  |  | Women (n=9) | 58.4% | 1.38 (0.29-6.44) |  |  |  |
|  | Race/Skin color | White (n=6) | 47.1% | -- | -- | -- | -- |
|  |  | Black (n=2) | 7.9% |  |  |  |  |
|  |  | Asian (n=0) * | -- |  |  |  |  |
|  |  | Brown/Mixed race (n=9) | 45.1% |  |  |  |  |
|  |  | Indigenous (n=0) * | -- |  |  |  |  |
|  | ***Adults*** | | | | | | |
|  | Schooling | 0-4 years (n=18) | 15.9% | 1 | 0.103 | 1 | 0.082 |
|  |  | 5-8 years (n=30) | 7.2% | 0.14 (0.02-0.83) |  | 0.12 (0.02-0.70) |  |
|  |  | 9-11 years (n=97) | 53.2% | 0.34 (0.14-0.81) |  | 0.33 (0.13-0.80) |  |
|  |  | 12 or more years (n=69) | 23.7% | 0.14 (0.03-0.72) |  | 0.13 (0.03-0.65) |  |
|  | Sex | Men (n=94) | 54.5% | 1 | 0.004 | 1 | **0.001** |
|  |  | Women (n=121) | 45.5% | 0.39 (0.21-0.74) |  | 0.37 (0.20-0.68) |  |
|  | Race/Skin color | White (n=58) | 39.6% | 1 | 0.102 | 1 | 0.175 |
|  |  | Black (n=38) | 10.4% | 0.85 (0.32-2.27) |  | 0.72 (0.27-1.96) |  |
|  |  | Asian (n=2) | 0.5% | 0.48 (0.06-3.90) |  | 0.42 (0.05-3.77) |  |
|  |  | Brown/Mixed race (n=111) | 48.2% | 1.32 (0.67-2.60) |  | 1.27 (0.60-2.67) |  |
|  |  | Indigenous (n=3) | 1.4% | 5.72 (1.33-24.64) |  | 7.56 (1.27-45.02) |  |
|  | ***Older adults*** | | | | | | |
|  | Schooling | 0-4 years (n=33) | 53.2% | 1 | 0.001 | 1 | **0.001** |
|  |  | 5-8 years (n=37) | 20.5% | 0.51 (0.18-1.45) |  | 0.51 (0.18-1.45) |  |
|  |  | 9-11 years (n=32) | 17% | 0.41 (0.15-1.12) |  | 0.41 (0.15-1.12) |  |
|  |  | 12 or more years (n=33) | 9.2% | 0.14 (0.05-0.39) |  | 0.14 (0.05-0.39) |  |
|  | Sex | Men (n=76) | 58.2% | 1 | 0.390 | -- | -- |
|  |  | Women (n=60) | 41.8% | 0.66 (0.26-1.70) |  |  |  |
|  | Race/Skin color | White (n=60) | 50.2% | -- | -- | -- | -- |
|  |  | Black (n=25) | 13.1% |  |  |  |  |
|  |  | Asian (n=2) | 1.7% |  |  |  |  |
|  |  | Brown/Mixed race (n=48) | 35% |  |  |  |  |
|  |  | Indigenous (n=0) * | -- |  |  |  |  |

ᵃ Odds ratio

ᵇ Confidence interval

* Category with no observations; model could not be estimated.

-- Not estimated or not applicable.

Estimates for small subgroups should be interpreted with caution due to limited sample size.

**Supplementary Table 2.** Associations between sociodemographic characteristics, including household crowding, and periodontal outcomes in the overall population and by age group (SB Brasil 2023).

| **Outcome** | **Sociodemographic variables** | **Categories** | **Weighted**  **(%)** | **Crude OR^a^**  **(95% CI^b^)** | **p-value** | **Adjusted OR**  **(95% CI)** | **Adjusted p-value** |
| --- | --- | --- | --- | --- | --- | --- | --- |
| Gingival signs | ***All ages*** | | | | | | |
|  | Schooling | 0-4 years (n=170) | 3.5% | 1 | 0.013 | 1 | 0.268 |
|  |  | 5-8 years (n=593) | 10% | 1.46 (0.86-2.49) |  | 1.10 (0.62-1.95) |  |
|  |  | 9-11 years (n=2446) | 51% | 2.22 (1.30-3.80) |  | 1.48 (0.82-2.68) |  |
|  |  | 12 or more years (n=1599) | 35.6% | 2.07 (1.20-3.54) |  | 1.50 (0.83-2.72) |  |
|  | Age range | Older adults (n=319) | 3.5% | 1 | <0.001 | 1 | **<0.001** |
|  |  | Adults (n=2388) | 65.4% | 3.32 (2.14-5.15) |  | 3.31 (1.97-5.60) |  |
|  |  | Adolescents (n=2119) | 31.2% | 4.01 (2.41-6.15) |  | 2.83 (1.82-4.41) |  |
|  | Sex | Men (n=2010) | 38.8% | 1 | 0.970 | -- | -- |
|  |  | Women (n=2816) | 61.2% | 1.00 (0.83-1.20) |  |  |  |
|  | Race/Skin color | White (n=1586) | 42.2% | 1 | 0.600 | -- | -- |
|  |  | Black (n=610) | 14.1% | 1.29 (0.79-2.09) |  |  |  |
|  |  | Asian (n=49) | 1.1% | 1.05 (0.48-2.31) |  |  |  |
|  |  | Brown/Mixed race (n=2524) | 42.4% | 1.07 (0.86-1.34) |  |  |  |
|  |  | Indigenous (n=14) | 0.2% | 0.60 (0.26-1.39) |  |  |  |
|  | Household crowding | Up to 3 people (n=1907) | 42.6% | 1 | 0.028 | 1 | 0.217 |
|  |  | 4 to 6 people (n=2642) | 54.1% | 1.31 (1.07-1.59) |  | 1.18 (0.98-1.42) |  |
|  |  | 7 or more people (n=277) | 3.3% | 1.19 (0.78-1.81) |  | 1.06 (0.69-1.62) |  |
|  | ***Adolescents*** | | | | | | |
|  | Sex | Men (n=1098) | 51.9% | 1 | 0.342 | -- | -- |
|  |  | Women (n=1021) | 48.1% | 0.85 (0.60-1.19) |  |  |  |
|  | Race/Skin color | White (n=649) | 42.2% | 1 | 0.866 | -- | -- |
|  |  | Black (n=242) | 14.1% | 1.16 (0.68-2.00) |  |  |  |
|  |  | Asian (n=21) | 1.1% | 0.75 (0.24-2.33) |  |  |  |
|  |  | Brown/Mixed race (n=1180) | 42.4% | 0.95 (0.66-1.37) |  |  |  |
|  |  | Indigenous (n=8) | 0.2% | 1.59 (0.17-14.96) |  |  |  |
|  | Household crowding | Up to 3 people (n=682) | 32.8% | 1 | 0.595 | -- | -- |
|  |  | 4 to 6 people (n=1280) | 61.7% | 1.01 (0.75-1.37) |  |  |  |
|  |  | 7 or more people (n=157) | 5.5% | 0.75 (0.41-1.37) |  |  |  |
|  | ***Adults*** | | | | | | |
|  | Schooling | 0-4 years (n=84) | 3.3% | 1 | 0.325 | -- | -- |
|  |  | 5-8 years (n=266) | 10.8% | 1.24 (0.56-2.75) |  |  |  |
|  |  | 9-11 years (n=860) | 42.8% | 1.80 (0.79-4.10) |  |  |  |
|  |  | 12 or more years (n=1168) | 43.2% | 1.66 (0.77-3.57) |  |  |  |
|  | Sex | Men (n=782) | 32.3% | 1 | 0.699 | -- | -- |
|  |  | Women (n=1606) | 67.4% | 1.05 (0.82-1.33) |  |  |  |
|  | Race/Skin color | White (n=791) | 42.4% | 1 | 0.374 | -- | -- |
|  |  | Black (n=318) | 15.4% | 1.31 (0.68-2.50) |  |  |  |
|  |  | Asian (n=25) | 1.2% | 1.35 (0.49-3.72) |  |  |  |
|  |  | Brown/Mixed race (n=1229) | 41% | 1.05 (0.75-1.48) |  |  |  |
|  |  | Indigenous (n=5) | 0.1% | 0.28 (0.07-1.16) |  |  |  |
|  | Household crowding | Up to 3 people (n=1005) | 45.5% | 1 | 0.246 | 1 | 0.246 |
|  |  | 4 to 6 people (n=1268) | 52.1% | 1.23 (0.94-1.61) |  | 1.23 (0.94-1.61) |  |
|  |  | 7 or more people (n=157) | 2.4% | 1.34 (0.75-2.39) |  | 1.34 (0.75-2.39) |  |
|  | ***Older adults*** | | | | | | |
|  | Schooling | 0-4 years (n=63) | 28.5% | 1 | 0.089 | 1 | 0.114 |
|  |  | 5-8 years (n=74) | 14.9% | 0.71 (0.27-1.91) |  | 0.71 (0.26-1.92) |  |
|  |  | 9-11 years (n=74) | 14.8% | 0.69 (0.27-1.79) |  | 0.71 (0.27-1.95) |  |
|  |  | 12 or more years (n=106) | 41.8% | 1.44 (0.47-4.45) |  | 1.43 (0.49-4.60) |  |
|  | Sex | Men (n=130) | 42.2% | 1 | 0.319 | -- | -- |
|  |  | Women (n=189) | 57.8% | 1.37 (0.74-2.53) |  |  |  |
|  | Race/Skin color | White (n=146) | 63.9% | 1 | 0.013 | 1 | **0.012** |
|  |  | Black (n=50) | 12.3% | 1.02 (0.51-2.05) |  | 1.00 (0.50-1.97) |  |
|  |  | Asian (n=3) | 0.1% | 0.06 (0.01-0.38) |  | 0.07 (0.01-0.37) |  |
|  |  | Brown/Mixed race (n=115) | 23.5% | 0.72 (0.29-1.82) |  | 0.74 (0.31-1.80) |  |
|  |  | Indigenous (n=1) | 0.3% | 3.59 (0.32-40.15) |  | 3.78 (0.32-44.99) |  |
|  | Household crowding | Up to 3 people (n=220) | 74.3% | 1 | 0.946 | -- | -- |
|  |  | 4 to 6 people (n=94) | 24.4% | 1.13 (0.56-2.28) |  |  |  |
|  |  | 7 or more people (n=5) | 1.4% | 1.10 (0.17-7.17) |  |  |  |
| Periodontal involvement | ***All ages*** | | | | | | |
|  | Schooling | 0-4 years (n=138) | 8.2% | 1 | <0.001 | 1 | 0.064 |
|  |  | 5-8 years (n=299) | 19.1% | 1.10 (0.52-2.30) |  | 1.40 (0.65-2.99) |  |
|  |  | 9-11 years (n=651) | 39.8% | 0.51 (0.28-0.94) |  | 0.92 (0.44-1.92) |  |
|  |  | 12 or more years (n=626) | 32.9% | 0.59 (0.25-1.39) |  | 0.72 (0.26-1.95) |  |
|  | Age range | Older adults (n=463) | 14.3% | 1 | <0.001 | 1 | **<0.001** |
|  |  | Adults (n=1039) | 79.2% | 0.66 (0.47-0.92) |  | 0.84 (0.48-1.46) |  |
|  |  | Adolescents (n=223) | 6.5% | 0.11 (0.07-0.17) |  | 0.12 (0.07-0.21) |  |
|  | Sex | Men (n=745) | 44.8% | 1 | 0.015 | 1 | **0.001** |
|  |  | Women (n=980) | 55.2% | 0.75 (0.59-0.95) |  | 0.65 (0.50-0.85) |  |
|  | Race/Skin color | White (n=605) | 46.3% | 1 | 0.153 | 1 | 0.149 |
|  |  | Black (n=266) | 13.8% | 1.05 (0.60-1.85) |  | 1.04 (0.56-1.93) |  |
|  |  | Asian (n=18) | 0.6% | 0.52 (0.19-1.40) |  | 0.56 (0.19-1.63) |  |
|  |  | Brown/Mixed race (n=816) | 38.6% | 0.85 (0.62-1.16) |  | 0.92 (0.66-1.26) |  |
|  |  | Indigenous (n=9) | 0.6% | 2.48 (0.87-7.05) |  | 2.85 (1.10-7.36) |  |
|  | Household crowding | Up to 3 people (n=863) | 53.2% | 1 | 0.187 | 1 | 0.937 |
|  |  | 4 to 6 people (n=772) | 44.5% | 0.74 (0.49-1.14) |  | 0.92 (0.59-1.45) |  |
|  |  | 7 or more people (n=90) | 2.3% | 0.56 (0.30-1.06) |  | 0.94 (0.50-1.77) |  |
|  | ***Adolescents*** | | | | | | |
|  | Sex | Men (n=119) | 61.1% | 1 | 0.108 | 1 | 0.071 |
|  |  | Women (n=104) | 38.9% | 0.61 (0.34-1.11) |  | 0.58 (0.32-1.05) |  |
|  | Race/Skin color | White (n=59) | 29% | 1 | 0.004 | 1 | **0.003** |
|  |  | Black (n=30) | 29% | 3.96 (1.64-9.56) |  | 4.11 (1.67-10.09) |  |
|  |  | Asian (n=4) | 2.6% | 3.34 (0.61-18.43) |  | 3.95 (0.75-20.72) |  |
|  |  | Brown/Mixed race (n=127) | 39.4% | 1.09 (0.56-2.13) |  | 1.10 (0.56-2.18) |  |
|  |  | Indigenous (n=1) | 0% | 0.15 (0.02-1.50) |  | 0.16 (0.02-1.52) |  |
|  | Household crowding | Up to 3 people (n=70) | 29.2% | 1 | 0.868 | -- | -- |
|  |  | 4 to 6 people (n=129) | 63.3% | 1.17 (0.59-2.32) |  |  |  |
|  |  | 7 or more people (n=24) | 7.5% | 1.30 (0.45-3.80) |  |  |  |
|  | ***Adults*** | | | | | | |
|  | Schooling | 0-4 years (n=48) | 6.1% | 1 | 0.123 | 1 | 0.141 |
|  |  | 5-8 years (n=160) | 18.8% | 1.14 (0.43-3.04) |  | 1.09 (0.44-2.70) |  |
|  |  | 9-11 years (n=400) | 40.9% | 0.71 (0.29-1.75) |  | 0.70 (0.29-1.67) |  |
|  |  | 12 or more years (n=429) | 34.2% | 0.53 (0.16-1.73) |  | 0.51 (0.16-1.63) |  |
|  | Sex | Men (n=403) | 42% | 1 | 0.004 | 1 | **0.005** |
|  |  | Women (n=636) | 58% | 0.62 (0.45-0.85) |  | 0.63 (0.45-0.87) |  |
|  | Race/Skin color | White (n=335) | 45.2% | 1 | 0.067 | 1 | 0.052 |
|  |  | Black (n=163) | 13.4% | 0.95 (0.50-1.97) |  | 0.89 (0.41-1.91) |  |
|  |  | Asian (n=9) | 0.4% | 0.31 (0.09-1.14) |  | 0.30 (0.08-1.09) |  |
|  |  | Brown/Mixed race (n=520) | 40.2% | 0.94 (0.65-1.36) |  | 0.87 (0.60-1.26) |  |
|  |  | Indigenous (n=7) | 0.8% | 3.23 (1.09-9.54) |  | 3.12 (1.07-9.15) |  |
|  | Household crowding | Up to 3 people (n=440) | 50.3% | 1 | 0.961 | -- | -- |
|  |  | 4 to 6 people (n=541) | 47.6% | 0.93 (0.55-1.58) |  |  |  |
|  |  | 7 or more people (n=58) | 2.1% | 0.96 (0.44-2.10) |  |  |  |
|  | ***Older adults*** | | | | | | |
|  | Schooling | 0-4 years (n=83) | 24.4% | 1 | 0.816 | -- | -- |
|  |  | 5-8 years (n=101) | 19.7% | 1.21 (0.53-2.77) |  |  |  |
|  |  | 9-11 years (n=111) | 22.7% | 1.43 (0.65-3.15) |  |  |  |
|  |  | 12 or more years (n=160) | 33.1% | 1.34 (0.63-2.85) |  |  |  |
|  | Sex | Men (n=223) | 52.8% | 1 | 0.353 | -- | -- |
|  |  | Women (n=240) | 47.2% | 0.81 (0.51-1.27) |  |  |  |
|  | Race/Skin color | White (n=211) | 60.1% | 1 | 0.933 | -- | -- |
|  |  | Black (n=73) | 9.3% | 0.76 (0.35-1.65) |  |  |  |
|  |  | Asian (n=5) | 1.2% | 0.81 (0.09-7.43) |  |  |  |
|  |  | Brown/Mixed race (n=169) | 29.3% | 1.01 (0.54-1.89) |  |  |  |
|  |  | Indigenous (n=1) | 0.1% | 1.50 (0.13-16.77) |  |  |  |
|  | Household crowding | Up to 3 people (n=353) | 80.5% | 1 | 0.159 | 1 | 0.159 |
|  |  | 4 to 6 people (n=102) | 19% | 0.74 (0.36-1.53) |  | 0.74 (0.36-1.53) |  |
|  |  | 7 or more people (n=8) | 0.5% | 0.32 (0.09-1.12) |  | 0.32 (0.09-1.12) |  |
| Severe periodontal involvement | ***All ages*** | | | | | | |
|  | Schooling | 0-4 years (n=52) | 21.6% | 1 | <0.001 | 1 | 0.076 |
|  |  | 5-8 years (n=70) | 9.8% | 0.18 (0.05-0.72) |  | 0.21 (0.05-0.89) |  |
|  |  | 9-11 years (n=142) | 47.9% | 0.23 (0.13-0.41) |  | 0.40 (0.19-0.86) |  |
|  |  | 12 or more years (n=102) | 20.8% | 0.14 (0.04-0.51) |  | 0.16 (0.04-0.68) |  |
|  | Age range | Older adults (n=136) | 17.7% | 1 | <0.001 | 1 | **<0.001** |
|  |  | Adults (n=215) | 79.7% | 0.56 (0.35-0.91) |  | 1.13 (0.48-2.66) |  |
|  |  | Adolescents (n=17) | 2.5% | 0.04 (0.02-0.10) |  | 0.06 (0.03-0.14) |  |
|  | Sex | Men (n=178) | 54.8% | 1 | 0.020 | 1 | **0.004** |
|  |  | Women (n=190) | 45.2% | 0.51 (0.29-0.90) |  | 0.42 (0.23-0.74) |  |
|  | Race/Skin color | White (n=124) | 41.7% | 1 | 0.222 | 1 | 0.214 |
|  |  | Black (n=65) | 10.8% | 0.90 (0.42-1.94) |  | 0.85 (0.39-1.85) |  |
|  |  | Asian (n=4) | 0.7% | 0.64 (0.14-2.90) |  | 0.74 (0.15-3.63) |  |
|  |  | Brown/Mixed race (n=168) | 45.7% | 1.15 (0.57-2.31) |  | 1.30 (0.65-2.62) |  |
|  |  | Indigenous (n=3) | 1.1% | 4.49 (1.11-18.19) |  | 6.54 (1.23-34.75) |  |
|  | Household crowding | Up to 3 people (n=201) | 59.1% | 1 | 0.023 | 1 | 0.460 |
|  |  | 4 to 6 people (n=151) | 39.5% | 0.61 (0.41-0.90) |  | 0.76 (0.47-1.22) |  |
|  |  | 7 or more people (n=680) | 1.4% | 0.32 (0.10-1.04) |  | 0.57 (0.16-2.03) |  |
|  | ***Adolescents*** | | | | | | |
|  | Sex | Men (n=8) | 41.6% | 1 | 0.684 | -- | -- |
|  |  | Women (n=9) | 58.4% | 1.38 (0.29-6.44) |  |  |  |
|  | Race/Skin color | White (n=6) | 47.1% | -- | -- | -- | -- |
|  |  | Black (n=2) | 7.9% |  |  |  |  |
|  |  | Asian (n=0) * | -- |  |  |  |  |
|  |  | Brown/Mixed race (n=9) | 45.1% |  |  |  |  |
|  |  | Indigenous (n=0) * | -- |  |  |  |  |
|  | Household crowding | Up to 3 people (n=3) | 5% | 1 | 0.045 | 1 | **0.045** |
|  |  | 4 to 6 people (n=10) | 90.1% | 9.62 (1.62-57.17) |  | 9.62 (1.62-57.17) |  |
|  |  | 7 or more people (n=4) | 4.8% | 4.84 (0.67-35.08) |  | 4.84 (0.67-35.08) |  |
|  | ***Adults*** | | | | | | |
|  | Schooling | 0-4 years (n=18) | 15.9% | 1 | 0.103 | 1 | 0.082 |
|  |  | 5-8 years (n=30) | 7.2% | 0.14 (0.02-0.83) |  | 0.12 (0.02-0.70) |  |
|  |  | 9-11 years (n=97) | 53.2% | 0.34 (0.14-0.81) |  | 0.33 (0.13-0.80) |  |
|  |  | 12 or more years (n=69) | 23.7% | 0.14 (0.03-0.72) |  | 0.13 (0.03-0.65) |  |
|  | Sex | Men (n=94) | 54.5% | 1 | 0.004 | 1 | **0.001** |
|  |  | Women (n=121) | 45.5% | 0.39 (0.21-0.74) |  | 0.37 (0.20-0.68) |  |
|  | Race/Skin color | White (n=58) | 39.6% | 1 | 0.102 | 1 | 0.175 |
|  |  | Black (n=38) | 10.4% | 0.85 (0.32-2.27) |  | 0.72 (0.27-1.96) |  |
|  |  | Asian (n=2) | 0.5% | 0.48 (0.06-3.90) |  | 0.42 (0.05-3.77) |  |
|  |  | Brown/Mixed race (n=111) | 48.2% | 1.32 (0.67-2.60) |  | 1.27 (0.60-2.67) |  |
|  |  | Indigenous (n=3) | 1.4% | 5.72 (1.33-24.64) |  | 7.56 (1.27-45.02) |  |
|  | Household crowding | Up to 3 people (n=96) | 55.6% | 1 | 0.482 | -- | -- |
|  |  | 4 to 6 people (n=109) | 43% | 0.76 (0.47-1.23) |  |  |  |
|  |  | 7 or more people (n=10) | 1.4% | 0.58 (0.16-2.15) |  |  |  |
|  | ***Older adults*** | | | | | | |
|  | Schooling | 0-4 years (n=33) | 53.2% | 1 | 0.001 | 1 | **0.001** |
|  |  | 5-8 years (n=37) | 20.5% | 0.51 (0.18-1.45) |  | 0.51 (0.18-1.45) |  |
|  |  | 9-11 years (n=32) | 17% | 0.41 (0.15-1.12) |  | 0.41 (0.15-1.12) |  |
|  |  | 12 or more years (n=33) | 9.2% | 0.14 (0.05-0.39) |  | 0.14 (0.05-0.39) |  |
|  | Sex | Men (n=76) | 58.2% | 1 | 0.390 | -- | -- |
|  |  | Women (n=60) | 41.8% | 0.66 (0.26-1.70) |  |  |  |
|  | Race/Skin color | White (n=60) | 50.2% | -- | -- | -- | -- |
|  |  | Black (n=25) | 13.1% |  |  |  |  |
|  |  | Asian (n=2) | 1.7% |  |  |  |  |
|  |  | Brown/Mixed race (n=48) | 35% |  |  |  |  |
|  |  | Indigenous (n=0) * | -- |  |  |  |  |
|  | Household crowding | Up to 3 people (n=102) | 82.5% | 1 | 0.401 | -- | -- |
|  |  | 4 to 6 people (n=32) | 16.9% | 0.67 (0.23-1.97) |  |  |  |
|  |  | 7 or more people (n=2) | 0.5% | 0.35 (0.07-1.80) |  |  |  |

ᵃ Odds ratio

ᵇ Confidence interval

* Category with no observations; model could not be estimated.

-- Not estimated or not applicable.

Estimates for small subgroups should be interpreted with caution due to limited sample size.

**Supplementary Table 3.** Weighted logistic regression models for fewer than 20 teeth (SB Brasil 2023).

| **Outcome** | **Sociodemographic variables** | **Categories**  **(n)** | **Weighted**  **(%)** | **Crude OR^a^**  **(95% CI^b^)** | **p-value** | **Adjusted OR**  **(95% CI)** | **Adjusted p-value** |
| --- | --- | --- | --- | --- | --- | --- | --- |
| < 20 teeth | ***All ages*** | | | | | | |
|  | Schooling | 0-4 years (n=2273) | 42% | 1 | <0.001 | 1 | **<0.001** |
|  |  | 5-8 years (n=1556) | 27.9% | 0.31 (0.22-0.44) |  | 0.63 (0.34-1.15) |  |
|  |  | 9-11 years (n=1170) | 19.5% | 0.06 (0.04-0.07) |  | 0.26 (0.15-0.44) |  |
|  |  | 12 or more years (n=756) | 10.6% | 0.04 (0.03-0.06) |  | 0.13 (0.07-0.22) |  |
|  | Age range | Older adults (n=5287) | 78.2% | 1 | <0.001 | 1 | **<0.001** |
|  |  | Adults (n=471) | 20.4% | 0.03 (0.02-0.05) |  | 0.05 (0.03-0.07) |  |
|  |  | Adolescents (n=81) | 1.4% | 0.01 (0.00-0.01) |  | 0.01 (0.00-0.02) |  |
|  | Sex | Men (n=2061) | 36.1% | 1 | 0.204 | -- | -- |
|  |  | Women (n=3778) | 63.9% | 1.12 (0.94-1.34) |  |  |  |
|  | Race/Skin color | White (n=1969) | 43.7% | 1 | 0.645 | -- | -- |
|  |  | Black (n=799) | 13.5% | 1.08 (0.81-1.43) |  |  |  |
|  |  | Asian (n=74) | 1.1% | 1.06 (0.55-2.03) |  |  |  |
|  |  | Brown/Mixed race (n=2889) | 41.1% | 0.98 (0.79-1.22) |  |  |  |
|  |  | Indigenous (n=33) | 0.6% | 2.17 (0.88-5.32) |  |  |  |
|  | ***Adolescents*** | | | | | | |
|  | Sex | Men (n=40) | 60.7% | 1 | 0.393 | -- | -- |
|  |  | Women (n=41) | 39.3% | 0.64 (0.22-1.80) |  |  |  |
|  | Race/Skin color | White (n=34) | 41.0% | -- |  | -- | -- |
|  |  | Black (n=9) | 19.9% |  |  |  |  |
|  |  | Asian (n=3) | 10.2% |  |  |  |  |
|  |  | Brown/Mixed race (n=35) | 28.9% |  |  |  |  |
|  |  | Indigenous (n=0) * | -- |  |  |  |  |
|  | ***Adults*** | | | | | | |
|  | Schooling | 0-4 years (n=91) | 30.0% | 1 | <0.001 | 1 | **<0.001** |
|  |  | 5-8 years (n=115) | 27.7% | 0.33 (0.13-0.86) |  | 0.33 (0.13-0.86) |  |
|  |  | 9-11 years (n=147) | 26.8% | 0.10 (0.05-0.23) |  | 0.10 (0.05-0.23) |  |
|  |  | 12 or more years (n=114) | 15.5% | 0.06 (0.02-0.13) |  | 0.06 (0.02-0.13) |  |
|  | Sex | Men (n=783) | 32.3% | 1 | 0.719 | -- | -- |
|  |  | Women (n=1612) | 67.7% | 0.89 (0.47-1.68) |  |  |  |
|  | Race/Skin color | White (n=128) | 34.8% | 1 | 0.308 | -- | -- |
|  |  | Black (n=68) | 11.9% | 1.11 (0.53-2.32) |  |  |  |
|  |  | Asian (n=8) | 1.5% | 1.89 (0.55-6.49) |  |  |  |
|  |  | Brown/Mixed race (n=262) | 51.5% | 1.58 (0.82-3.04) |  |  |  |
|  |  | Indigenous (n=3) | 0.3% | 1.24 (0.28-5.53) |  |  |  |
|  | ***Older adults*** | | | | | | |
|  | Schooling | 0-4 years (n=2181) | 45.7% | 1 | <0.001 | 1 | **<0.001** |
|  |  | 5-8 years (n=1430) | 28.3% | 0.88 (0.57-1.37) |  | 0.88 (0.57-1.37) |  |
|  |  | 9-11 years (n=973) | 16.9% | 0.52 (0.30-0.90) |  | 0.50 (0.29-0.87) |  |
|  |  | 12 or more years (n=623) | 9.1% | 0.18 (0.12-0.28) |  | 0.18 (0.12-0.27) |  |
|  | Sex | Men (n=1894) | 35.8% | 1 | <0.001 | 1 | **<0.001** |
|  |  | Women (n=3393) | 64.2% | 1.71 (1.32-2.22) |  | 1.96 (1.47-2.60) |  |
|  | Race/Skin color | White (n=1807) | 46.0% | 1 | 0.002 | 1 | 0.104 |
|  |  | Black (n=722) | 13.7% | 1.58 (0.99-2.51) |  | 1.28 (0.79-2.06) |  |
|  |  | Asian (n=63) | 0.9% | 0.80 (0.21-3.05) |  | 0.64 (0.14-2.86) |  |
|  |  | Brown/Mixed race (n=2592) | 38.6% | 1.73 (1.30-2.31) |  | 1.42 (1.02-1.98) |  |
|  |  | Indigenous (n=30) | 0.7% | 9.81 (2.96-32.49) |  | 10.38 (2.22-48.57) |  |

ᵃ Odds ratio

ᵇ Confidence interval

* Category with no observations; model could not be estimated.

-- Not estimated or not applicable.

Estimates for small subgroups should be interpreted with caution due to limited sample size.

.

**Supplementary Table 4.** Weighted logistic regression analyses of periodontal outcomes in the full sample, regardless of the number of teeth (SB Brasil 2023).

| **Outcome** | **Sociodemographic variables** | **Categories**  **(n)** | **Weighted**  **(%)** | **Crude OR^a^**  **(95% CI^b^)** | **p-value** | **Adjusted OR**  **(95% CI)** | **Adjusted p-value** |
| --- | --- | --- | --- | --- | --- | --- | --- |
| Gingival signs | ***All ages*** | | | | | | |
|  | Schooling | 0-4 years (n=175) | 3.5% | 1 | <0.001 | 1 | **0.004** |
|  |  | 5-8 years (n=593) | 10% | 2.75 (1.66-4.55) |  | 1.51 (0.89-2.56) |  |
|  |  | 9-11 years (n=2448) | 51% | 6.49 (4.05-10.41) |  | 2.33 (1.36-3.98) |  |
|  |  | 12 or more years (n=1602) | 35.6% | 6.27 (3.90-10.06) |  | 2.47 (1.42-4.30) |  |
|  | Age range | Older adults (n=322) | 3.5% | 1 | <0.001 | 1 | **<0.001** |
|  |  | Adults (n=2395) | 65.4% | 11.23 (7.15-17.62) |  | 10.72 (6.31-18.20) |  |
|  |  | Adolescents (n=2119) | 31.1% | 15.01 (8.95-25.18) |  | 8.38 (5.27-13.31) |  |
|  | Sex | Men (n=2013) | 38.7% | 1 | 0.761 | -- | -- |
|  |  | Women (n=2823) | 61.3% | 0.97 (0.82-1.15) |  |  |  |
|  | Race/Skin color | White (n=1586) | 42.2% | 1 | 0.603 | -- | -- |
|  |  | Black (n=611) | 14.1% | 1.23 (0.78-1.93) |  |  |  |
|  |  | Asian (n=49) | 1.1% | 0.95 (0.42-2.13) |  |  |  |
|  |  | Brown/Mixed race (n=2533) | 42.4% | 1.07 (0.87-1.34) |  |  |  |
|  |  | Indigenous (n=14) | 0.2% | 0.49 (0.23-1.04) |  |  |  |
|  | ***Adolescents*** | | | | | | |
|  | Sex | Men (n=1098) | 51.9% | 1 | 0.358 | -- | -- |
|  |  | Women (n=1021) | 48.1% | 0.85 (0.61-1.20) |  |  |  |
|  | Race/Skin color | White (n=649) | 39.4% | 1 | 0.873 | -- | -- |
|  |  | Black (n=242) | 11.7% | 1.14 (0.65-2.01) |  |  |  |
|  |  | Asian (n=21) | 0.9% | 0.65 (0.22-1.94) |  |  |  |
|  |  | Brown/Mixed race (n=1235) | 41.0% | 0.96 (0.67-1.38) |  |  |  |
|  |  | Indigenous (n=5) | 0.2% | 1.63 (0.17-15.38) |  |  |  |
|  | ***Adults*** | | | | | | |
|  | Schooling | 0-4 years (n=86) | 3.3% | 1 | 0.009 | 1 | **0.009** |
|  |  | 5-8 years (n=266) | 10.7% | 1.69 (0.84-3.39) |  | 1.69 (0.84-3.39) |  |
|  |  | 9-11 years (n=862) | 42.8% | 2.79 (1.33-5.86) |  | 2.79 (1.33-5.86) |  |
|  |  | 12 or more years (n=1171) | 43.1% | 2.67 (1.36-5.24) |  | 2.67 (1.36-5.24) |  |
|  | Sex | Men (n=783) | 32.3% | 1 | 0.609 | -- | -- |
|  |  | Women (n=1612) | 67.7% | 1.07 (0.83-1.38) |  |  |  |
|  | Race/Skin color | White (n=791) | 42.3% | 1 | 0.678 | -- | -- |
|  |  | Black (n=319) | 15.3% | 1.29 (0.68-2.43) |  |  |  |
|  |  | Asian (n=25) | 1.2% | 1.04 (0.35-3.08) |  |  |  |
|  |  | Brown/Mixed race (n=1235) | 41% | 1.00 (0.71-1.07) |  |  |  |
|  |  | Indigenous (n=5) | 0.1% | 0.28 (0.07-1.07) |  |  |  |
|  | ***Older adults*** | | | | | | |
|  | Schooling | 0-4 years (n=66) | 28.6% | 1 | 0.004 | 1 | **0.007** |
|  |  | 5-8 years (n=74) | 14.9% | 0.81 (0.28-2.35) |  | 0.79 (0.26-2.36) |  |
|  |  | 9-11 years (n=74) | 14.7% | 1.17 (0.49-2.81) |  | 1.15 (0.46-2.91) |  |
|  |  | 12 or more years (n=106) | 41.8% | 4.22 (1.35-13.17) |  | 3.93 (1.17-13.17) |  |
|  | Sex | Men (n=132) | 42.2% | 1 | 0.734 | -- | -- |
|  |  | Women (n=190) | 57.8% | 0.90 (0.48-1.68) |  |  |  |
|  | Race/Skin color | White (n=146) | 63.8% | 1 | 0.123 | 1 | 0.269 |
|  |  | Black (n=50) | 12.2% | 0.73 (0.40-1.32) |  | 0.82 (0.44-1.54) |  |
|  |  | Asian (n=3) | 0.1% | 0.78 (0.04-0.17) |  | 0.08 (0.03-0.16) |  |
|  |  | Brown/Mixed race (n=118) | 23.6% | 0.50 (0.22-1.15) |  | 0.60 (0.26-1.37) |  |
|  |  | Indigenous (n=1) | 0.3% | 0.36 (0.13-1.02) |  | 0.52 (0.20-1.39) |  |
| Periodontal involvement | ***All ages*** | | | | | | |
|  | Schooling | 0-4 years (n=141) | 8.3% | 1 | 0.152 | 1 | 0.316 |
|  |  | 5-8 years (n=304) | 19.2% | 2.12 (1.07-4.21) |  | 1.84 (0.87-3.91) |  |
|  |  | 9-11 years (n=655) | 39.7% | 1.55 (0.92-2.62) |  | 1.50 (0.72-3.13) |  |
|  |  | 12 or more years (n=630) | 32.9% | 1.84 (0.84-4.04) |  | 1.30 (0.49-3.40) |  |
|  | Age range | Older adults (n=473) | 14.4% | 1 | <0.001 | 1 | **<0.001** |
|  |  | Adults (n=1045) | 79.1% | 2.51 (1.77-3.55) |  | 2.61 (1.50-4.55) |  |
|  |  | Adolescents (n=223) | 6.5% | 0.44 (0.29-0.68) |  | 0.40 (0.23-0.70) |  |
|  | Sex | Men (n=751) | 44.8% | 1 | 0.010 | 1 | **0.001** |
|  |  | Women (n=990) | 55.2% | 0.74 (0.59-0.93) |  | 0.61 (0.48-0.78) |  |
|  | Race/Skin color | White (n=606) | 46.2% | 1 | 0.515 | -- |  |
|  |  | Black (n=269) | 13.8% | 1.04 (0.59-1.82) |  |  |  |
|  |  | Asian (n=19) | 0.6% | 0.49 (0.19-1.27) |  |  |  |
|  |  | Brown/Mixed race (n=827) | 38.7% | 0.86 (0.63-1.16) |  |  |  |
|  |  | Indigenous (n=9) | 0.6% | 1.78 (0.63-5.03) |  |  |  |
|  | ***Adolescents*** | | | | | | |
|  | Sex | Men (n=119) | 61.1% | 1 | 0.099 | 1 | 0.064 |
|  |  | Women (n=104) | 38.9% | 0.62 (0.35-1.10) |  | 0.59 (0.34-1.03) |  |
|  | Race/Skin color | White (n=59) | 29% | 1 | 0.003 | 1 | **0.002** |
|  |  | Black (n=30) | 29% | 3.91 (1.65-9.30) |  | 4.02 (1.68-9.65) |  |
|  |  | Asian (n=4) | 2.6% | 3.00 (0.54-16.51) |  | 3.57 (0.69-18.54) |  |
|  |  | Brown/Mixed race (n=127) | 39.4% | 1.10 (0.54-2.22) |  | 1.11 (0.54-2.25) |  |
|  |  | Indigenous (n=1) | 0% | 0.15 (0.04-0.52) |  | 0.16 (0.05-0.51) |  |
|  | ***Adults*** | | | | | | |
|  | Schooling | 0-4 years (n=48) | 6.1% | 1 | 0.330 | -- |  |
|  |  | 5-8 years (n=162) | 18.7% | 1.55 (0.54-4.48) |  |  |  |
|  |  | 9-11 years (n=402) | 40.8% | 1.11 (0.45-2.74) |  |  |  |
|  |  | 12 or more years (n=431) | 34.4% | 0.87 (0.27-2.84) |  |  |  |
|  | Sex | Men (n=403) | 41.9% | 1 | 0.005 | 1 | **0.005** |
|  |  | Women (n=642) | 58.1% | 0.64 (0.46-0.87) |  | 0.64 (0.46-0.87) |  |
|  | Race/Skin color | White (n=336) | 45.3% | 1 | 0.511 | -- |  |
|  |  | Black (n=163) | 13.4% | 0.94 (0.45-1.99) |  |  |  |
|  |  | Asian (n=9) | 0.4% | 0.26 (0.07-0.95) |  |  |  |
|  |  | Brown/Mixed race (n=525) | 40.2% | 0.90 (0.63-1.29) |  |  |  |
|  |  | Indigenous (n=7) | 0.8% | 3.05 (1.03-9.03) |  |  |  |
|  | ***Older adults*** | | | | | | |
|  | Schooling | 0-4 years (n=86) | 24.7% | 1 | <0.001 | 1 | **<0.001** |
|  |  | 5-8 years (n=104) | 20.2% | 1.30 (0.64-2.66) |  | 1.32 (0.66-2.64) |  |
|  |  | 9-11 years (n=113) | 22.5% | 2.18 (0.95-4.99) |  | 2.28 (0.99-5.23) |  |
|  |  | 12 or more years (n=162) | 32.6% | 3.92 (1.94-7.91) |  | 4.10 (2.06-8.13) |  |
|  | Sex | Men (n=228) | 53.3% | 1 | 0.003 | 1 | **0.001** |
|  |  | Women (n=244) | 46.7% | 0.55 (0.37-0.81) |  | 0.51 (0.34-0.76) |  |
|  | Race/Skin color | White (n=211) | 59.2% | 1 | 0.274 | -- | -- |
|  |  | Black (n=76) | 9.8% | 0.61 (0.30-1.26) |  |  |  |
|  |  | Asian (n=6) | 1.2% | 0.98 (0.21-4.62) |  |  |  |
|  |  | Brown/Mixed race (n=175) | 29.7% | 0.68 (0.39-1.21) |  |  |  |
|  |  | Indigenous (n=1) | 0.1% | 0.18 (0.02-1.73) |  |  |  |
| Severe periodontal involvement | ***All ages*** | | | | | | |
|  | Schooling | 0-4 years (n=53) | 21.5% | 1 | 0.342 | -- |  |
|  |  | 5-8 years (n=71) | 9.8% | 0.49 (0.15-1.68) |  |  |  |
|  |  | 9-11 years (n=145) | 47.9% | 1.11 (0.72-1.70) |  |  |  |
|  |  | 12 or more years (n=103) | 20.8% | 0.69 (0.20-2.35) |  |  |  |
|  | Age range | Older adults (n=139) | 17.8% | 1 | <0.001 | 1 | **<0.001** |
|  |  | Adults (n=218) | 79.7% | 3.35 (1.88-5.99) |  | 3.61 (1.99-6.56) |  |
|  |  | Adolescents (n=17) | 2.5% | 0.23 (0.10-0.56) |  | 0.21 (0.09-0.53) |  |
|  | Sex | Men (n=179) | 54.7% | 1 | 0.020 | 1 | **0.002** |
|  |  | Women (n=195) | 45.3% | 0.48 (0.28-0.82) |  | 0.39 (0.22-0.69) |  |
|  | Race/Skin color | White (n=124) | 41.7% | 1 | 0.008 | 1 | 0.644 |
|  |  | Black (n=65) | 10.8% | 0.90 (0.42-1.94) |  | 0.92 (0.44-1.94) |  |
|  |  | Asian (n=4) | 0.7% | 0.64 (0.14-2.90) |  | 0.51 (0.11-2.44) |  |
|  |  | Brown/Mixed race (n=168) | 45.7% | 1.15 (0.57-2.31) |  | 1.16 (0.56-2.50) |  |
|  |  | Indigenous (n=3) | 1.1% | 4.49 (1.11-18.19) |  | 3.62 (0.85-15.36) |  |
|  | ***Adolescents*** | | | | | | |
|  | Sex | Men (n=8) | 41.6% | 1 | 0.776 | -- | -- |
|  |  | Women (n=9) | 58.4% | 1.25 (0.27-5.80) |  |  |  |
|  | Race/Skin color | White (n=6) | 47.1% | -- |  | -- | -- |
|  |  | Black (n=2) | 7.9% |  |  |  |  |
|  |  | Asian (n=0) * | -- |  |  |  |  |
|  |  | Brown/Mixed race (n=9) | 45.1% |  |  |  |  |
|  |  | Indigenous (n=0) * | -- |  |  |  |  |
|  | ***Adults*** | | | | | | |
|  | Schooling | 0-4 years (n=18) | 15.9% | 1 | 0.134 | 1 | 0.118 |
|  |  | 5-8 years (n=31) | 7.2% | 0.28 (0.05-1.50) |  | 0.29 (0.06-1.54) |  |
|  |  | 9-11 years (n=98) | 53.1% | 0.81 (0.36-1.83) |  | 1.02 (0.48-2.20) |  |
|  |  | 12 or more years (n=70) | 23.8% | 0.32 (0.06-1.69) |  | 0.37 (0.07-1.82) |  |
|  | Sex | Men (n=94) | 54.4% | 1 | 0.003 | 1 | **0.002** |
|  |  | Women (n=124) | 45.6% | 0.37 (0.20-0.71) |  | 0.35 (0.18-0.67) |  |
|  | Race/Skin color | White (n=58) | 39.5% | 1 | 0.553 | -- |  |
|  |  | Black (n=38) | 10.4% | 0.95 (0.37-2.44) |  |  |  |
|  |  | Asian (n=2) | 0.4% | 0.33 (0.04-2.63) |  |  |  |
|  |  | Brown/Mixed race (n=114) | 48.3% | 1.23 (0.60-2.51) |  |  |  |
|  |  | Indigenous (n=3) | 1.4% | 4.79 (1.12-20.49) |  |  |  |
|  | ***Older adults*** | | | | | | |
|  | Schooling | 0-4 years (n=34) | 53.1% | 1 | 0.507 | 1 | 0.507 |
|  |  | 5-8 years (n=37) | 20.4% | 0.61 (0.23-1.64) |  | 0.61 (0.23-1.64) |  |
|  |  | 9-11 years (n=34) | 17.4% | 0.79 (0.24-1.39) |  | 0.79 (0.24-1.39) |  |
|  |  | 12 or more years (n=33) | 9.2% | 0.14 (0.05-0.39) |  | 0.14 (0.05-0.39) |  |
|  | Sex | Men (n=77) | 58.1% | 1 | 0.085 | -- | -- |
|  |  | Women (n=62) | 41.9% | 0.45 (0.18-1.12) |  |  |  |
|  | Race/Skin color | White (n=60) | 50.0% | -- | -- | -- | -- |
|  |  | Black (n=25) | 13.1% |  |  |  |  |
|  |  | Asian (n=2) | 1.7% |  |  |  |  |
|  |  | Brown/Mixed race (n=51) | 35.2% |  |  |  |  |
|  |  | Indigenous (n=0) * | -- |  |  |  |  |

ᵃ Odds ratio

ᵇ Confidence interval

* Category with no observations; model could not be estimated.

-- Not estimated or not applicable.

Estimates for small subgroups should be interpreted with caution due to limited sample size.

**Supplementary Table 5.** Fully adjusted weighted Poisson regression models for gingival signs, periodontal involvement, and severe periodontal involvement among individuals with ≥20 natural teeth (SB Brasil 2023).

| **Outcome** | **Sociodemographic variables** | **Categories**  **(n)** | **Weighted**  **(%)** | **Crude PR^a^**  **(95% CI^b^)** | **p-value** | **Adjusted PR**  **(95% CI)** | **Adjusted p-value** |
| --- | --- | --- | --- | --- | --- | --- | --- |
| Gingival signs | ***All ages*** | | | | | | |
|  | Schooling | 0-4 years (n=175) | 3.5% | 1 | 0.018 | 1 | 0.315 |
|  |  | 5-8 years (n=593) | 10% | 1.32 (0.89-1.98) |  | 1.08 (0.71-1.65) |  |
|  |  | 9-11 years (n=2448) | 51% | 1.74 (1.17-2.58) |  | 1.31 (0.85-2.01) |  |
|  |  | 12 or more years (n=1602) | 35.6% | 1.65 (1.11-2.48) |  | 1.31 (0.85-2.03) |  |
|  | Age range | Older adults (n=322) | 3.5% | 1 | <0.001 | 1 | **<0.001** |
|  |  | Adults (n=2395) | 65.4% | 2.45 (1.71-3.52) |  | 2.26 (1.55-3.29) |  |
|  |  | Adolescents (n=2119) | 31.1% | 2.75 (1.83-4.12) |  | 2.51 (1.64-3.84) |  |
|  | Sex | Men (n=2013) | 38.7% | 1 | 0.969 | -- | -- |
|  |  | Women (n=2823) | 61.3% | 1.00 (0.89-1.12) |  |  |  |
|  | Race/Skin color | White (n=1586) | 42.2% | 1 | 0.623 | -- | -- |
|  |  | Black (n=611) | 14.1% | 1.17 (0.88-1.55) |  |  |  |
|  |  | Asian (n=49) | 1.1% | 1.03 (0.63-1.71) |  |  |  |
|  |  | Brown/Mixed race (n=2533) | 42.4% | 1.05 (0.91-1.21) |  |  |  |
|  |  | Indigenous (n=14) | 0.2% | 0.70 (0.37-1.31) |  |  |  |
|  | ***Adolescents*** | | | | | | |
|  | Sex | Men (n=1098) | 51.9% | 1 | 0.336 | -- | -- |
|  |  | Women (n=1021) | 48.1% | 0.91 (0.75-1.11) |  |  |  |
|  | Race/Skin color | White (n=649) | 39.4% | 1 | 0.852 | -- | -- |
|  |  | Black (n=242) | 11.7% | 1.09 (0.80-1.47) |  |  |  |
|  |  | Asian (n=21) | 0.9% | 0.84 (0.40-1.74) |  |  |  |
|  |  | Brown/Mixed race (n=1235) | 41.0% | 0.97 (0.78-1.20) |  |  |  |
|  |  | Indigenous (n=5) | 0.2% | 1.27 (0.45-3.62) |  |  |  |
|  | ***Adults*** | | | | | | |
|  | Schooling | 0-4 years (n=86) | 3.3% | 1 | 0.347 | -- | -- |
|  |  | 5-8 years (n=266) | 10.7% | 1.17 (0.66-2.07) |  |  |  |
|  |  | 9-11 years (n=862) | 42.8% | 1.48 (0.83-2.64) |  |  |  |
|  |  | 12 or more years (n=1171) | 43.1% | 1.41 (0.81-2.44) |  |  |  |
|  | Sex | Men (n=783) | 32.3% | 1 | 0.699 | -- | -- |
|  |  | Women (n=1612) | 67.7% | 1.03 (0.89-1.20) |  |  |  |
|  | Race/Skin color | White (n=791) | 42.3% | 1 | 0.457 | -- | -- |
|  |  | Black (n=319) | 15.3% | 1.18 (0.81-1.72) |  |  |  |
|  |  | Asian (n=25) | 1.2% | 1.20 (0.67-2.14) |  |  |  |
|  |  | Brown/Mixed race (n=1235) | 41% | 1.03 (0.83-1.28) |  |  |  |
|  |  | Indigenous (n=5) | 0.1% | 0.38 (0.11-1.28) |  |  |  |
|  | ***Older adults*** | | | | | | |
|  | Schooling | 0-4 years (n=66) | 28.6% | 1 | 0.081 | 1 | 0.111 |
|  |  | 5-8 years (n=74) | 14.9% | 0.74 (0.32-1.75) |  | 0.74 (0.32-1.75) |  |
|  |  | 9-11 years (n=74) | 14.7% | 0.72 (0.32-1.65) |  | 0.75 (0.32-1.78) |  |
|  |  | 12 or more years (n=106) | 41.8% | 1.35 (0.54-3.40) |  | 1.34 (0.52-3.49) |  |
|  | Sex | Men (n=132) | 42.2% | 1 | 0.320 | -- | -- |
|  |  | Women (n=190) | 57.8% | 0.30 (0.77-2.20) |  |  |  |
|  | Race/Skin color | White (n=146) | 63.8% | 1 | 0.008 | 1 | **0.009** |
|  |  | Black (n=50) | 12.2% | 0.01 (0.57-1.82) |  | 1.00 (0.56-1.78) |  |
|  |  | Asian (n=3) | 0.1% | 0.72 (0.01-0.43) |  | 0.08 (0.01-0.42) |  |
|  |  | Brown/Mixed race (n=118) | 23.6% | 0.76 (0.34-1.67) |  | 0.78 (0.37-1.65) |  |
|  |  | Indigenous (n=1) | 0.3% | 2.51 (0.58-10.81) |  | 2.59 (0.58-11.53) |  |
| Periodontal involvement | ***All ages*** | | | | | | |
|  | Schooling | 0-4 years (n=141) | 8.3% | 1 | <0.001 | 1 | **0.042** |
|  |  | 5-8 years (n=304) | 19.2% | 1.07 (0.61-1.90) |  | 1.29 (0.72-2.29) |  |
|  |  | 9-11 years (n=655) | 39.7% | 0.57 (0.37-0.91) |  | 0.94 (0.53-1.65) |  |
|  |  | 12 or more years (n=630) | 32.9% | 0.65 (0.33-1.28) |  | 0.77 (0.35-1.66) |  |
|  | Age range | Older adults (n=473) | 14.4% | 1 | <0.001 | 1 | **<0.001** |
|  |  | Adults (n=1045) | 79.1% | 0.14 (0.09-0.21) |  | 0.86 (0.58-1.27) |  |
|  |  | Adolescents (n=223) | 6.5% | 0.44 (0.29-0.68) |  | 0.15 (0.10-0.24) |  |
|  | Sex | Men (n=751) | 44.8% | 1 | 0.016 | 1 | **0.001** |
|  |  | Women (n=990) | 55.2% | 0.78 (0.64-0.96) |  | 0.71 (0.58-0.87) |  |
|  | Race/Skin color | White (n=606) | 46.2% | 1 | 0.079 | 1 | 0.061 |
|  |  | Black (n=269) | 13.8% | 1.04 (0.64-1.71) |  | 1.02 (0.60-1.74) |  |
|  |  | Asian (n=19) | 0.6% | 0.56 (0.22-1.41) |  | 0.61 (0.23-1.61) |  |
|  |  | Brown/Mixed race (n=827) | 38.7% | 0.87 (0.66-1.14) |  | 0.93 (0.71-1.21) |  |
|  |  | Indigenous (n=9) | 0.6% | 1.99 (0.96-4.13) |  | 2.09 (1.12-3.90) |  |
|  | ***Adolescents*** | | | | | | |
|  | Sex | Men (n=119) | 61.1% | 1 | 0.102 | 1 | 0.066 |
|  |  | Women (n=104) | 38.9% | 0.62 (0.35-1.10) |  | 0.60 (0.34-1.04) |  |
|  | Race/Skin color | White (n=59) | 29% | 1 | 0.003 | 1 | **0.002** |
|  |  | Black (n=30) | 29% | 3.67 (1.60-8.40) |  | 3.78 (1.63-8.74) |  |
|  |  | Asian (n=4) | 2.6% | 3.14 (0.64-15.40) |  | 3.67 (0.79-17.09) |  |
|  |  | Brown/Mixed race (n=127) | 39.4% | 1.09 (0.54-2.19) |  | 1.10 (0.54-2.22) |  |
|  |  | Indigenous (n=1) | 0% | 0.15 (0.02-1.52) |  | 0.16 (0.02-1.54) |  |
|  | ***Adults*** | | | | | | |
|  | Schooling | 0-4 years (n=48) | 6.1% | 1 | 0.099 | 1 | 0.113 |
|  |  | 5-8 years (n=162) | 18.7% | 1.10 (0.53-2.28) |  | 1.06 (0.56-2.03) |  |
|  |  | 9-11 years (n=402) | 40.8% | 0.76 (0.39-1.49) |  | 0.76 (0.41-1.42) |  |
|  |  | 12 or more years (n=431) | 34.4% | 0.60 (0.25-1.48) |  | 0.59 (0.25-1.39) |  |
|  | Sex | Men (n=403) | 41.9% | 1 | 0.004 | 1 | **0.005** |
|  |  | Women (n=642) | 58.1% | 0.68 (0.52-0.88) |  | 0.69 (0.53-0.90) |  |
|  | Race/Skin color | White (n=336) | 45.3% | 1 | 0.037 | 1 | **0.029** |
|  |  | Black (n=163) | 13.4% | 0.96 (0.52-1.78) |  | 0.91 (0.48-1.71) |  |
|  |  | Asian (n=9) | 0.4% | 0.36 (0.11-1.23) |  | 0.35 (0.10-1.19) |  |
|  |  | Brown/Mixed race (n=525) | 40.2% | 0.95 (0.70-1.29) |  | 0.89 (0.66-1.21) |  |
|  |  | Indigenous (n=7) | 0.8% | 2.24 (1.17-4.30) |  | 2.16 (1.13-4.13) |  |
|  | ***Older adults*** | | | | | | |
|  | Schooling | 0-4 years (n=86) | 24.7% | 1 | 0.817 | -- | -- |
|  |  | 5-8 years (n=104) | 20.2% | 1.15 (0.60-2.21) |  |  |  |
|  |  | 9-11 years (n=113) | 22.5% | 1.31 (0.71-2.41) |  |  |  |
|  |  | 12 or more years (n=162) | 32.6% | 1.25 (0.70-2.23) |  |  |  |
|  | Sex | Men (n=228) | 53.3% | 1 | 0.344 | -- | -- |
|  |  | Women (n=244) | 46.7% | 0.85 (0.61-1.19) |  |  |  |
|  | Race/Skin color | White (n=211) | 59.2% | 1 | 0.934 | -- | -- |
|  |  | Black (n=76) | 9.8% | 0.82 (0.45-1.49) |  |  |  |
|  |  | Asian (n=6) | 1.2% | 0.85 (0.15-4.70) |  |  |  |
|  |  | Brown/Mixed race (n=175) | 29.7% | 1.00 (0.63-1.59) |  |  |  |
|  |  | Indigenous (n=1) | 0.1% | 1.32 (0.27-6.35) |  |  |  |
| Severe periodontal involvement | ***All ages*** | | | | | | |
|  | Schooling | 0-4 years (n=53) | 21.5% | 1 | <0.001 | 1 | 0.051 |
|  |  | 5-8 years (n=71) | 9.8% | 0.21 (0.06-0.70) |  | 0.24 (0.07-0.87) |  |
|  |  | 9-11 years (n=145) | 47.9% | 0.26 (0.17-0.41) |  | 0.45 (0.24-0.84) |  |
|  |  | 12 or more years (n=103) | 20.8% | 0.16 (0.05-0.50) |  | 0.18 (0.05-0.64 |  |
|  | Age range | Older adults (n=139) | 17.8% | 1 | <0.001 | 1 | **<0.001** |
|  |  | Adults (n=218) | 79.7% | 0.59 (0.37-0.93) |  | 1.07 (0.48-2.35) |  |
|  |  | Adolescents (n=17) | 2.5% | 0.04 (0.02-0.11) |  | 0.06 (0.03-0.13) |  |
|  | Sex | Men (n=179) | 54.7% | 1 | 0.018 | 1 | **0.001** |
|  |  | Women (n=195) | 45.3% | 0.52 (0.30-0.90) |  | 0.44 (0.27-0.73) |  |
|  | Race/Skin color | White (n=124) | 41.7% | 1 | 0.198 | 1 | 0.116 |
|  |  | Black (n=65) | 10.8% | 0.91 (0.42-1.96) |  | 0.83 (0.39-1.77) |  |
|  |  | Asian (n=4) | 0.7% | 0.64 (0.15-2.85) |  | 0.72 (0.16-3.29) |  |
|  |  | Brown/Mixed race (n=168) | 45.7% | 1.14 (0.58-2.24) |  | 1.25 (0.68-2.30) |  |
|  |  | Indigenous (n=3) | 1.1% | 3.96 (1.18-13.29) |  | 4.81 (1.39-16.69) |  |
|  | ***Adolescents*** | | | | | | |
|  | Sex | Men (n=8) | 41.6% | 1 | 0.688 | -- | -- |
|  |  | Women (n=9) | 58.4% | 1.38 (0.29-6.55) |  |  |  |
|  | Race/Skin color | White (n=6) | 47.1% | -- | -- | -- | -- |
|  |  | Black (n=2) | 7.9% |  |  |  |  |
|  |  | Asian (n=0) * | -- |  |  |  |  |
|  |  | Brown/Mixed race (n=9) | 45.1% |  |  |  |  |
|  |  | Indigenous (n=0) * | -- |  |  |  |  |
|  | ***Adults*** | | | | | | |
|  | Schooling | 0-4 years (n=18) | 15.9% | 1 | 0.046 | 1 | **0.026** |
|  |  | 5-8 years (n=31) | 7.2% | 0.16 (0.03-0.76) |  | 0.14 (0.03-0.63) |  |
|  |  | 9-11 years (n=98) | 53.1% | 0.38 (0.19-0.75) |  | 0.38 (0.20-0.73) |  |
|  |  | 12 or more years (n=70) | 23.8% | 0.16 (0.04-0.66) |  | 0.16 (0.04-0.59) |  |
|  | Sex | Men (n=94) | 54.4% | 1 | 0.003 | 1 | **<0.001** |
|  |  | Women (n=124) | 45.6% | 0.41 (0.23-0.74) |  | 0.40 (0.24-0.67) |  |
|  | Race/Skin color | White (n=58) | 39.5% | 1 | 0.059 | 1 | 0.076 |
|  |  | Black (n=38) | 10.4% | 0.85 (0.32-2.27) |  | 0.74 (0.29-1.88) |  |
|  |  | Asian (n=2) | 0.4% | 0.49 (0.06-3.82) |  | 0.45 (0.06-3.65) |  |
|  |  | Brown/Mixed race (n=114) | 48.3% | 1.30 (0.68-2.48) |  | 1.25 0.63-2.48) |  |
|  |  | Indigenous (n=3) | 1.4% | 4.72 (1.42-15.68) |  | 5.54 (1.47-20.95) |  |
|  | ***Older adults*** | | | | | | |
|  | Schooling | 0-4 years (n=34) | 53.1% | 1 | 0.003 | 1 | **0.003** |
|  |  | 5-8 years (n=37) | 20.4% | 0.55 (0.22-1.39) |  | 0.55 (0.22-1.39) |  |
|  |  | 9-11 years (n=34) | 17.4% | 0.45 (0.19-1.07) |  | 0.45 (0.19-1.07) |  |
|  |  | 12 or more years (n=33) | 9.2% | 0.16 (0.06-0.44) |  | 0.16 (0.06-0.44) |  |
|  | Sex | Men (n=77) | 58.1% | 1 | 0.394 | -- | -- |
|  |  | Women (n=62) | 41.9% | 0.69 (0.29-1.64) |  |  |  |
|  | Race/Skin color | White (n=60) | 50.0% | -- | -- | -- | -- |
|  |  | Black (n=25) | 13.1% |  |  |  |  |
|  |  | Asian (n=2) | 1.7% |  |  |  |  |
|  |  | Brown/Mixed race (n=51) | 35.2% |  |  |  |  |
|  |  | Indigenous (n=0) * | -- |  |  |  |  |

ᵃ Prevalence ratio

ᵇ Confidence interval

* Category with no observations; model could not be estimated.

-- Not estimated or not applicable.

Estimates for small subgroups should be interpreted with caution due to limited sample size.

**Supplementary Table 6.** Weighted prevalences and temporal changes in periodontal outcomes between 2010 and 2023 according to sociodemographic characteristics (2023-2010).

| **Outcome** | **Sociodemographic variables** | **Categories** | **Weighted %**  **(95% CI^a^)**  **2010** | **Weighted %**  **(95% CI)**  **2023** | **Prevalence difference (p.p.)**  **(95% CI)**  **2010-2023** | **p-value** |
| --- | --- | --- | --- | --- | --- | --- |
| Gingival signs | Age range | Adolescents | 38.34 (34.42-41.87) | 41.91 (37.35-46.60) | 3.83  (-2.15 to 9.82) | 0.209 |
|  |  | Adults | 30.34 (27.58-33.25) | 37.39 (32.98-42.02) | 7.51  (1.68 to 12.43) | **0.010** |
|  |  | Older adults | 17.11 (11.90-24.00) | 15.24 (10.49-21.63) | -1.87  (-10.04 to 6.29) | 0.653 |
|  | Sex | Men | 33.99 (30.42-37.75) | 36.72 (32.72-40.90) | 2.72  (-2.80 to 8.26) | 0.334 |
|  |  | Women | 33.02 (30.36-35.80) | 36.80 (32.58-41.23) | 3.78  (-1.38 to 8.93) | 0.151 |
|  | Race/Skin color | White | 31.97 (29.25-34.82) | 35.34 (31.39-39.50) | 3.37  (-1.59 to 8.33) | 0.183 |
|  |  | Black | 28.65 (23.51-34.40) | 41.28 (29.58-54.06) | 12.64  (-1.00 to 26.28) | 0.069 |
|  |  | Asian | 21.14 (12.26-31.98) | 36.51 (21.22-55.11) | 15.36  (-4.45 to 35.17) | 0.128 |
|  |  | Brown/Mixed race | 36.98 (32.87-41.29) | 36.97 (33.01-41.11) | -0.01  (-5.91 to 5.88) | 0.997 |
|  |  | Indigenous | 43.67 (23.53-66.15) | 24.70 (12.48-43.01) | -18.97  (-46.21 to 8.26) | 0.172 |
|  | Schooling | 0-4 years | 26.74 (21.69-32.48) | 22.82 (15.50-32.26) | -3.92  (-13.89 to 6.05) | 0.440 |
|  |  | 5-8 years | 35.66 (31.99-39.51) | 30.22 (24.45-36.68) | -5.45  (-12.64 to 1.75) | 0.138 |
|  |  | 9-11 years | 35.10 (31.31-39.10) | 39.63 (33.96-45.59) | 4.53  (-2.51 to 11.57) | 0.207 |
|  |  | 12 or more years | 31.38 (26.48-36.73) | 37.80 (33.26-42.57) | 6.43  (-0.54 to 13.39) | 0.070 |
|  | Household crowding | Up to 3 people | 30.63 (27.50-33.96) | 33.52 (29.45-37.85) | 2.89  (-2.44 to 8.21) | 0.288 |
|  |  | 4 to 6 people | 34.73 (31.28-38.36) | 39.75 (35.21-44.47) | 5.02  (-0.86 to 10.89) | 0.094 |
|  |  | 7 or more people | 35.82 (30.28-41.77) | 37.49 (29.10-46.70) | 1.67  (-8.94 to 12.27) | 0.758 |
|  | Total population | | 33.44 (30.92-36.06) | 36.77 (33.14-40.56) | 3.32  (-1.23 to 7.88) | 0.152 |
| Periodontal involvement | Age range | Adolescents | 9.49 (7.44-12.02) | 3.65 (2.61-5.09) | -5.83  (-8.42 to -3.25) | **<0.001** |
|  |  | Adults | 33.37 (29.77-37.17) | 18.95 (15.11-23.50) | -14.41  (-20.02 to -8.81) | **<0.001** |
|  |  | Older adults | 32.71 (25.40-40.98) | 26.20 (20.39-32.97) | -6.52  (-16.57 to -3.54) | 0.204 |
|  | Sex | Men | 23.72 (20.69-27.05) | 17.74 (14.00-22.21) | -5.99  (-11.18 to -0.79) | **0.024** |
|  |  | Women | 20.78 (17.95-23.92) | 13.88 (11.17-17.11) | -6.90  (-11.06 to -2.71) | **0.001** |
|  | Race/Skin color | White | 21.53 (18.56-24.83) | 16.34 (12.20-21.54) | -5.19  (-10.80 to 0.42) | 0.070 |
|  |  | Black | 24.84 (18.30-32.77) | 17.04 (11.92-23.75) | -7.80  (-17.14 to 1.54) | 0.102 |
|  |  | Asian | 18.21 (10.80-29.03) | 9.17 (3.72-20.87) | -9.03  (-20.97 to 2.90) | 0.138 |
|  |  | Brown/Mixed race | 22.20 (18.84-25.95) | 14.16 (10.98-18.07) | -8.04  (-13.05 to -3.02) | **0.002** |
|  |  | Indigenous | 17.50 (8.31-33.17) | 32.58 (15.18-56.62) | 15.08  (-9.71 to 39.88) | 0.233 |
|  | Schooling | 0-4 years | 31.02 (25.96-36.59) | 22.32 (11.27-39.38) | -8.70  (-23.79 to 6.39) | 0.258 |
|  |  | 5-8 years | 25.33 (21.63-29.42) | 23.95 (17.13-32.41) | -1.38  (-9.97 to 7.20) | 0.752 |
|  |  | 9-11 years | 17.82 (14.81-21.29) | 12.83 (9.62-16.91) | -4.99  (-9.85 to -0.13) | **0.044** |
|  |  | 12 or more years | 18.87 (15.32-25.38) | 14.50 (11.91-17.53) | -5.38  (-11.11 to 0.36) | 0.066 |
|  | Household crowding | Up to 3 people | 24.83 (21.42-28.58) | 17.54 (12.62-23.84) | -7.29  (-13.92 to -0.66) | **0.031** |
|  |  | 4 to 6 people | 20.16 (17.27-23.40) | 13.67 (11.23-16.53) | -6.49  (-10.54 to -2.45) | **0.002** |
|  |  | 7 or more people | 23.80 (18.33-30.31) | 10.68 (6.67-16.67) | -13.13  (-20.87 to -5.38) | **0.001** |
|  | Total population | | 22.06 (19.50-24.85) | 15.38 (12.56-18.69) | -6.68  (-10.75 to -2.62) | **0.001** |
| Severe periodontal involvement | Age range | Adolescents | 0.62 (0.32-1.19) | 0.37 (0.17-0.82) | -0.25  (-0.75 to 0.25) | 0.328 |
|  |  | Adults | 9.14 (7.26-11.45) | 4.98 (2.68-9.06) | -4.17  (-7.86 to -0.47) | **0.027** |
|  |  | Older adults | 13.53 (8.98-19.89) | 8.50 (5.41-13.11) | -5.04  (-11.60 to 1.53) | 0.133 |
|  | Sex | Men | 5.93 (4.17-8.36) | 5.67 (2.64-11.74) | -0.26  (-4.98 to 4.47) | 0.915 |
|  |  | Women | 4.82 (3.64-6.36) | 2.96 (2.03-4.31) | -1.86  (-3.61 to -0.11) | **0.038** |
|  | Race/Skin color | White | 5.52 (4.13-7.36) | 3.82 (2.32-6.21) | -1.71  (-4.18 to 0.76) | 0.175 |
|  |  | Black | 5.50 (3.61-8.28) | 3.46 (2.04-5.81) | -2.04  (-4.94 to 0.86) | 0.169 |
|  |  | Asian | 5.34 (2.37-11.58) | 2.46 (0.60-9.44) | -2.88  (-8.33 to 2.56) | 0.299 |
|  |  | Brown/Mixed race | 5.02 (3.71-6.77) | 4.35 (1.79-10.21) | -0.67  (-4.78 to 3.43) | 0.748 |
|  |  | Indigenous | 1.48 (0.45-4.75) | 15.10 (4.59-39.66) | 13.62  (-3.22 to 30.46) | 0.113 |
|  | Schooling | 0-4 years | 10.62 (7.47-14.89) | 15.18 (5.36-36.14) | 4.56  (-10.72 to 19.83) | 0.558 |
|  |  | 5-8 years | 6.80 (4.98-9.22) | 3.16 (1.90-5.24) | -3.63  (-6.28 to -0.99) | **0.007** |
|  |  | 9-11 years | 3.48 (2.44-4.94) | 4.00 (1.99-7.87) | 0.52  (-2.50 to 3.53) | 0.736 |
|  |  | 12 or more years | 3.36 (1.62-6.82) | 2.37 (1.31-4.24) | -0.99  (-3.77 to 1.80) | 0.487 |
|  | Household crowding | Up to 3 people | 6.38 (4.84-8.36) | 5.08 (2.71-9.31) | -1.30  (-4.89 to 2.30) | 0.479 |
|  |  | 4 to 6 people | 4.66 (3.54-6.12) | 3.17 (1.87-5.32) | -1.49  (-3.58 to 0.60) | 0.163 |
|  |  | 7 or more people | 5.32 (2.50-10.97) | 1.68 (0.64-4.37) | -3.64  (-7.90 to 0.62) | 0.092 |
|  | Total population | | 5.30 (4.24-6.61) | 4.01 (2.31-6.89) | -1.29  (-3.78 to 1.21) | 0.311 |

ᵃ Confidence interval
